# Phylogenetic relationships in *Cortinarius*, section *Calochroi*, inferred from nuclear DNA sequences

**DOI:** 10.1186/1471-2148-9-1

**Published:** 2009-01-02

**Authors:** Sigisfredo Garnica, Michael Weiß, Bernhard Oertel, Joseph Ammirati, Franz Oberwinkler

**Affiliations:** 1 Lehrstuhl für Spezielle Botanik und Mykologie, Botanisches Institut, Universität Tübingen, Auf der Morgenstelle 1, D-72076 Tübingen, Germany; 2 IRES Gartenbauwissenschaft, Universität Bonn, Auf dem Hügel 6, D-53121 Bonn, Germany; 3 Department of Biology, Box 355325, University of Washington, Seattle, Washington 98195-5325, USA

## Abstract

**Background:**

Section *Calochroi *is one of the most species-rich lineages in the genus *Cortinarius *(Agaricales, Basidiomycota) and is widely distributed across boreo-nemoral areas, with some extensions into meridional zones. Previous phylogenetic studies of *Calochroi *(incl. section *Fulvi*) have been geographically restricted; therefore, phylogenetic and biogeographic relationships within this lineage at a global scale have been largely unknown. In this study, we obtained DNA sequences from a nearly complete taxon sampling of known species from Europe, Central America and North America. We inferred intra- and interspecific phylogenetic relationships as well as major morphological evolutionary trends within section *Calochroi *based on 576 ITS sequences, 230 ITS + 5.8S + D1/D2 sequences, and a combined dataset of ITS + 5.8S + D1/D2 and RPB1 sequences of a representative subsampling of 58 species.

**Results:**

More than 100 species were identified by integrating DNA sequences with morphological, macrochemical and ecological data. *Cortinarius *section *Calochroi *was consistently resolved with high branch support into at least seven major lineages: *Calochroi*, *Caroviolacei*, *Dibaphi*, *Elegantiores*, *Napi*, *Pseudoglaucopodes *and *Splendentes*; whereas *Rufoolivacei *and *Sulfurini *appeared polyphyletic. A close relationship between *Dibaphi*, *Elegantiores*, *Napi *and *Splendentes *was consistently supported. Combinations of specific morphological, pigmentation and molecular characters appear useful in circumscribing clades.

**Conclusion:**

Our analyses demonstrate that *Calochroi *is an exclusively northern hemispheric lineage, where species follow their host trees throughout their natural ranges within and across continents. Results of this study contribute substantially to defining European species in this group and will help to either identify or to name new species occurring across the northern hemisphere. Major groupings are in partial agreement with earlier morphology-based and molecular phylogenetic hypotheses, but some relationships were unexpected, based on external morphology. In such cases, their true affinities appear to have been obscured by the repeated appearance of similar features among distantly related species. Therefore, further taxonomic studies are needed to evaluate the consistency of species concepts and interpretations of morphological features in a more global context. Reconstruction of ancestral states yielded two major evolutionary trends within section *Calochroi*: (1) the development of bright pigments evolved independently multiple times, and (2) the evolution of abruptly marginate to flattened stipe bulbs represents an autapomorphy of the *Calochroi *clade.

## Background

*Calochroi *is one of the most diverse lineages within the *Cortinarius *radiation, with estimates of between 90 and 170 species recognised so far from Europe [1], which probably represents only a portion of the taxa on a global scale. All members of this section are obligate root symbionts (ectomycorrhizal); therefore, the presence of appropriate plant hosts as well as specific edaphic factors play a crucial role in their geographical distributions. In general, the highest interspecific diversity roughly matches the distribution of coniferous (*Abies*, *Picea*) and broadleaf (*Fagus*, *Quercus*, *Lithocarpus*) trees across boreo-nemoral areas. Often, shared ecological specialisations makes it possible to find basidiomes of different species associated with an individual host tree on the same site. Section *Calochroi*, as circumscribed here, comprises species producing conspicuous basidiomes with a rounded to adruptly bulbous stipe base, a pileus with a well-developed gelatinous layer, and the presence of pigments which often change colour with the application of KOH. Most species produce basidiomes after the first autumn rains; however, in North America, certain species with a persistent veil, short stipe and subemerged basidiomes occur in spring. In oak forests of Costa Rica, a number of species occur during the rainy season in June, and sometimes later in the season as well. Traditionally, species concepts have been largely based on macromorphological traits, colour changes with KOH on the basidiomes (usually the pileus, and the stipe surface and flesh), and ecological specialisations [2-5]. However, some of these traits are heavily labile and/or affected by homoplasy, and as a result, the taxonomy of this group is extremely difficult, and much controversy exists in the delimitation and recognition of species. Because of their abundance in many forest ecosystems, and the spectacular appearance and colouration of the basidiomes, calochroid species have been the focus of numerous taxonomic studies [see, for example [2-6]], as well as the chemical structures of their pigments [7-11]. Most recently, considerable progress has been made in reconstructing phylogenetic relationships among European calochroid species through analyses of morphological features and DNA sequences. Peintner et al. [12] studied the phylogeny of sequestrate taxa related to *Cortinarius *based on ITS sequences and distinguished a well-supported lineage named *Phlegmacium *IV containing a small number of European and North American calochroid species. Garnica et al. [13] analysed the phylogenetic relationships of 54 European *Phlegmacium *species using ITS and D1/D2 sequences of the nuclear gene coding for the ribosomal large subunit (nucLSU rDNA) and found that members of the section *Calochroi *are distributed into two closely related lineages that can be assigned to *Calochroi *and *Fulvi*. Peintner et al. [14] and Garnica et al. [15] used a more extensive sampling of taxa across the genus *Cortinarius *and supported the monophyly of *Calochroi*. At least five subclades were distinguished by Peintner et al. [15]. Frøslev et al. [16] used 54 European phlegmacioid taxa and compared phylogenies resulting from RPB1, RPB2 and ITS data as single and combined data sets, and accepted three subclades named /*Calochroid*, /*Rufoolivacei *and /*Fulvi*. These authors showed that certain morphological features, such as a pileipellis structure (*pileipellis simplex*), a very broad marginate bulbous stipe base and coarse, net-like basidiospore ornamentation have evolved independently several times within the genus *Cortinarius*. Frøslev et al. [1] evaluated species delimitations using a total of 421 ITS sequences (including sequences from 53 type specimens) and recognised */Arcuatorum, /Calochroid, /Fulvi, /Rufoolivacei *and */Sulfurinus *as major subclades within section *Calochroi *in Europe. In several cases, these authors found that sequences obtained from collections of the same species from different geographical areas showed very little variation. Nevertheless, little is known about intra- and interspecific patterns of distribution and phylogenetic relationships within section *Calochroi *at a global scale. Morphology-based taxonomic studies have recorded *Cortinarius *species displaying calochroid and fulvoid features from several coniferous and frondose forests in North America [17,18] and *Nothofagus *forests in South America [19,20]. Furthermore, in light of similar morphological features of the basidiomes some calochroid species have been reported with European and North American disjunct distribution patterns [see [21-25]]. Therefore, to gain a more complete understanding of intra- and interspecific patterns of distribution and phylogenetic relationships at a global scale, we have attempted to use a more extensive sampling of calochroid and fulvoid *Phlegmacium *species, including European, North American, Tasmanian, New Zealand, and Central and South American collections.

Here, we present phylogenetic hypotheses including nearly all of the known species of *Calochroi sensu *M.M. Moser using nuclear DNA sequence data from the internal transcribed spacers (ITS1 and ITS2, including the 5.8S), the D1/D2 regions of the nucLSU rDNA and from the gene coding for domains A-C of RPB1 from a representative subsample. We use these phylogenies to address the following main objectives. First, we compared our own sequences with available ITS sequences from GenBank and UNITE databases to establish congruence and uniformity of species concepts for European calochroid species. Second, we estimated intra- and interspecific sequence divergences of closely related taxa by comparing the internal transcribed spacers (ITS1, ITS2) alone or including D1/D2 regions. Third, we inferred phylogenetic relationships and major morphological evolutionary trends within the section *Calochroi *by integrating molecular phylogenetic analyses of single and combined DNA sequence datasets with morphological, chemical and ecological features. This study provides the first insights into the geographical structure of species and populations occurring in parts of Central America, North America and Europe within a diverse and complex lineage of ectomycorrhizal fungi.

## Results

### Phylogenetic delimitation of the section *Calochroi*

**Figure 1:**
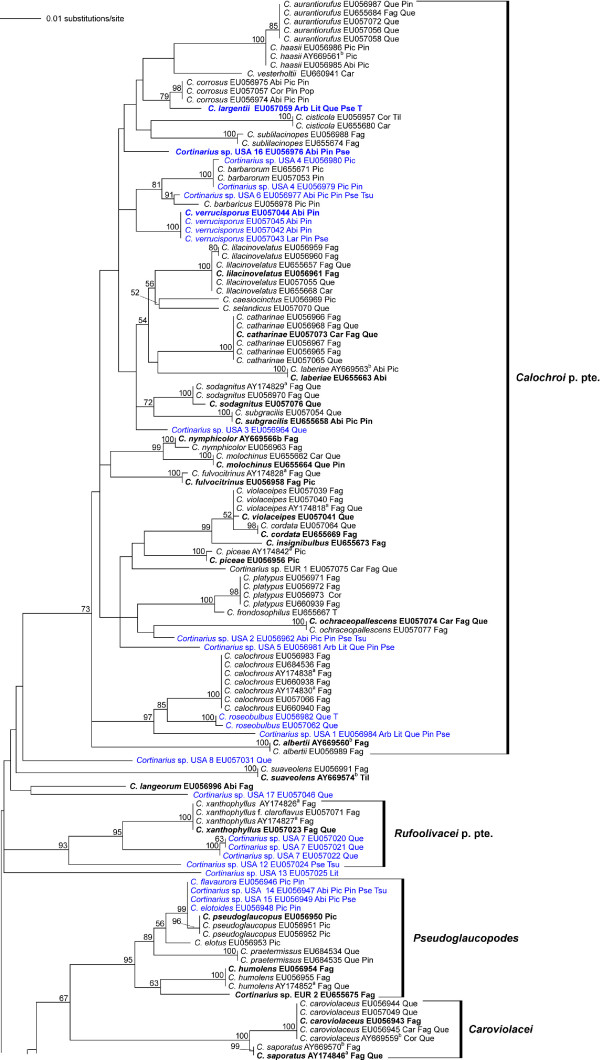
**Phylogenetic relationships within *Cortinarius *section *Calochroi *as derived from ML analysis of ITS-5.8S + D1/D2 rDNA sequences**. Numbers above branches are bootstrap values (values < 50% not shown) from 1000 replicates. North American *Calochroi *collections are printed in blue. Host tree genera are coded as follows: Betulaceae, Bet = *Betula*, Car = *Carpinus*, Cor = *Corylus*, Ericaceae, Arb = *Arbutus*; Fagaceae, Fag = *Fagus*, Que = *Quercus*, Lit = *Lithocarpus*; Pinaceae, Abi = *Abies*, Lar = *Larix*, Pic = *Picea*, Pin = *Pinus*, Pse = *Pseudotsuga*, Tsu = *Tsuga*; Salicaceae, Pop = *Populus*, Sal = *Salix*. Names in boldface correspond to sequences also used in the combined phylogenetic analysis (Figure 3); type collections are indicated by (T).

**Figure 2:**
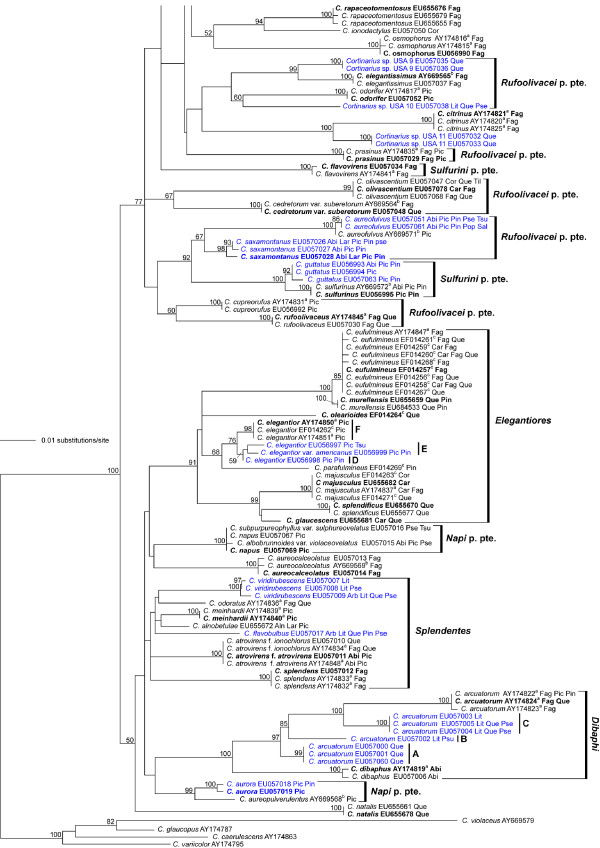
**Phylogenetic relationships within *Cortinarius *section *Calochroi *as derived from ML analysis of ITS-5.8S + D1/D2 rDNA sequences**. Numbers above branches are bootstrap values (values < 50% not shown) from 1000 replicates. North American *Calochroi *collections are printed in blue. Host tree genera are coded as follows: Betulaceae, Bet = *Betula*, Car = *Carpinus*, Cor = *Corylus*, Ericaceae, Arb = *Arbutus*; Fagaceae, Fag = *Fagus*, Que = *Quercus*, Lit = *Lithocarpus*; Pinaceae, Abi = *Abies*, Lar = *Larix*, Pic = *Picea*, Pin = *Pinus*, Pse = *Pseudotsuga*, Tsu = *Tsuga*; Salicaceae, Pop = *Populus*, Sal = *Salix*. Names in boldface correspond to sequences also used in the combined phylogenetic analysis (Figure 3); type collections are indicated by (T).

**Figure 3:**
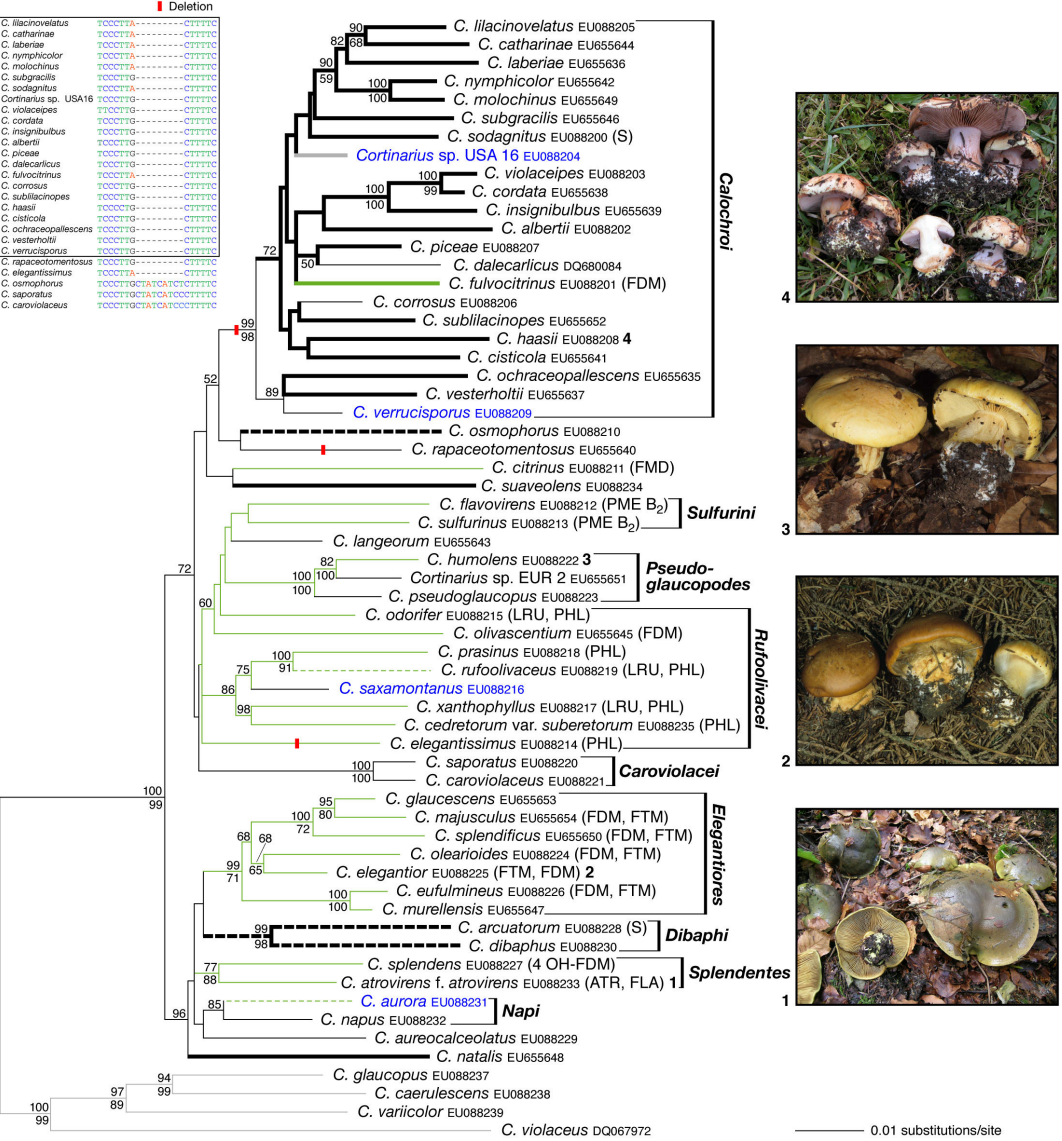
**Phylogeny of the section *Calochroi *as derived from ML analysis of an alignment of ITS-5.8S + D1/D2 (except for *C. dalecarlicus*) + RPB1 A-C sequences**. Numbers above nodes are ML bootstrap values, numbers below nodes are MP bootstrap values (values < 50% not shown) from 1000 replicates, respectively. Distributions of basidiome pigments were retrieved from [7,9,10,64] and are abbreviated as follows. Flavomannin pigments: ATR = atrovirin, FDM = flavomannin-6-6'dimethylether, 4-OH-FDM = 4-OH-flavomannin-6,6'-dimethylether, FLA = flavomannin, FTM = flavomannin-6,6',8-trimethylether; phlegmacin pigments: LRU = leucorufoolivacins, PHL = phlegmacin, PME B_2 _= phlegmacin-8'-methylether B_2_; triterpenoid pigments: S = sodagnitins A-F. Note that FDM is a common pigment found also in other groups in *Cortinarius*. A non-coding 8 bp insertion is absent in all species of the *Calochroi *clade and also in *C. elegantissimus *and *C. rapaceotomentosus*, but is present in the remaining species. The alignment shown includes all species of the *Calochroi *clade contained in the combined dataset 3 (framed box), and corresponds to positions 769–777 of intron 2 of the RPB1 sequence. Ancestral character states were reconstructed using maximum parsimony (DELTRAN strategy). Green branches indicate taxa of bright colours; taxa with abruptly marginate to flattened stipe bulbs are indicated by bold lines. Dashed branches indicate taxa where bright basidiome colours or marginate to flattened stipe bulbs are not continuously present in the populations. *C. elegantior *and *C. humolens *photos, credit: F. Röger.

The section *Calochroi *appeared as a well-supported monophyletic clade within the genus *Cortinarius *in our preliminary neighbor-joining analyses (tree not shown). These analyses also showed that *C. inexpectatus *from Europe, and *C. rhodophyllus*, *C. chlorophanus*, *C. stephanopus *and *C. vaginatus *from South America, which are currently classified in *Calochroi *and *Fulvi*, cluster outside of the section *Calochroi *as conceived here. Thus, all species assignable to section *Calochroi *(incl. section *Fulvi*) in this study are from the northern hemisphere. A total of 576 sequences including ITS and 5.8S, 230 sequences comprising ITS, 5.8S and nucLSU D1/D2 rDNA, and 56 RPB1 sequences including the conserved domains A-C were analysed in three separate datasets: dataset 1 including ITS and 5.8S sequences (Additional file 1); dataset 2 including ITS, 5.8S and D1/D2 sequences (Figures 1 and 2); dataset 3 including combined ITS, 5.8S and D1/D2, and RPB1 A-C sequences (Figure 3). The final sampling covered c. 110 species with geographical distributions across Europe, Central America and North America.

### Intraspecific variation and relationships between closely related taxa as inferred from ITS-5.8S and ITS-5.8S-D1/D2 sequences

#### ITS-5.8S sequences

Comparative intraspecific ITS-5.8S sequence analyses between our collections and those of Frøslev et al. [1,16] revealed identical sequences in *C. albertii*, *C. arcuatorum*, *C. barbaricus*, *C. cupreorufus*, *C. elegantior*, *C. langeorum*, *C. meinhardii, C. napus*, *C. odoratus*, *C. osmophorus*, *C. pseudoglaucopus*, *C. selandicus*, *C. subgracilis*, *C. sublilacinopes*, *C. sulfurinus *and *C. olivascentium*; whereas, for the remaining species, an intraspecific genetic variation of up to 6 bp was observed (Additional file 1). However, it should be noted that within the same species, GenBank sequences have deviating sites across relatively conservative portions of ITS1 and ITS2 regions compared to the sequences obtained in this study. In general, the species concepts used in the present work agree with those applied by Frøslev et al. [1,16] and Peintner et al. [12,14,26] (see Additional file 1). However, for nomenclatural reasons, we prefer to use the following names: *C. cedretorum *var. *cedretorum *(= *C. cedretorum*), *C. eufulmineus *(= *C. quercus-ilicis*), *C. majusculus *(= *C. alcalinophilus*), *C. alnobetulae *(= *C. moseri*) and *C. olivascentium *(= *C. xanthochlorus*).

#### ITS-5.8S-D1/D2 sequences

Many collections of the same species from different, but geographically restricted, localities (i.e. from populations in Central America, North America or Europe) shared identical sequences. Generally, sequence variation within different collections of the same species was caused by obvious polymorphic sites and/or single nucleotide indels distributed mainly across the ITS1 and ITS2 regions. There was a 0.1% sequence divergence between *C. albobrunnoides *var. *violaceovelatus *and *C. napus*; *C. albobrunnoides *var. *violaceovelatus *and *C. subpurpureophyllus *var. *sulphureovelatus*; and between different collections of *C. aureocalceolatus*, *C. aureofulvus *and *Cortinarius *sp. USA 7. Collections of *C. aurantiorufus *and *C. haasii, C. elotoides *and *C. pseudoglaucopus*, and *C. flavaurora *and *C. pseudoglaucopus *showed a 0.2% sequence divergence. There was a 0.2% sequence divergence within collections of *C. dibaphus *and *C. lilacinovelatus*, respectively. New World collections of *C. arcuatorum *clustered within three distinctive lineages designated as groups A-C in Figures 1 and 2: group A comprised the collections JFA 12037, JFA 12039 and JFA 12061 from Costa Rica with identical sequences; group B included the collection JFA 11893 from Mendocino, California, occupying an isolated position; and group C was composed of the collections JFA 11765, JFA 11766 and JFA 11803 from the Smith River drainage, Del Norte County, California, with identical DNA sequences. There was a 1.6% sequence divergence between group A and B, while group A and C differed by 1.5%, and group B and C differed by 2.1%. There were between 34–39 bp differences (approximately 2.5–2.8% sequence divergence) between New World and European collections of *C. arcuatorum*. New World and European *Elegantiores *collections were distributed into three monophyletic subgroups labeled as D-F in Figures 1 and 2: group D consists of *C. elegantior *var. *americanus *JFA 11452 from Wyoming with an isolated position; group E comprises the *C. elegantior *collections JFA 12438 from Wyoming and JFA 11693 from Oregon with a sequence divergence of 0.3%; and group F includes only European collections with identical sequences. Sequence divergences ranged from 0.2% to 0.5% among the groups F and D, and 0.6% to 0.7% between the groups D-E and D. Sequences from the following specimens were identical: *C. albobrunnoides *JFA 12426, *C. napus *and *C. subpurpureophyllus *var. *sulphureovelatus*; *C. claroflavus *and *C. xanthophyllus*; *C. elotoides *(IB 19870060 Holotypus), *Cortinarius *sp. JFA 11618 and JFA 11619 and *C. flavaurora*; *C. elotus *IB 1999/0192 and *C. pseudoglaucopus*; and *C. olivellus *and *C. humolens*. Collections of *C. aurora*, *C. corrosus *(TUB 012705, TUB 012706) and *C. rufoolivaceus *differed by single nucleotide indels in the ITS1 region, the collections TUB 012714 and TUB 012715 of *C. haasii *differed by a single nucleotide indel in the ITS2 region. Collections of *Cortinarius *sp. JFA 11833, JFA 11845 and *C. prasinus *had identical sequences and inclusively shared a polymorphism at the same position (Y = C/T).

### Phylogenetic reconstruction inferred from ITS, 5.8S and D1/D2 rDNA

Our phylogenetic analyses of dataset 2 (Figures 1 and 2) supported the monophyly of the clades *Calochroi*, *Caroviolacei*, *Dibaphi*, *Elegantiores*, *Splendentes *and *Pseudoglaucopodes*. ML bootstrap support for the monophyly of *Napi*, *Rufoolivacei *and *Sulfurini *was below 50%; these groups appear polyphyletic in the best tree found in heuristic ML analysis (Figures 1 and 2). *Cortinarius *spp. USA 8, USA 13 and USA 17, *C. aureocalceolatus*, *C. aureofulvus*, *C. ionodactylus*, *C. langeorum*, *C. natalis*, *C. osmophorus*, *C. rapaceotomentosus *and *C suaveolens *could not be assigned to particular clades. More than 35% of the species analysed were nested within the *Calochroi *clade; the remaining taxa were distributed over various smaller lineages. The partially hypogeous taxa clustered in the clades *Calochroi *and *Rufoolivacei*, respectively.

### Phylogenetic relationships inferred from ITS-5.8S-D1/D2 sequences combined with RPB1 domains A-C

Maximum likelihood and maximum parsimony analyses of dataset 3 revealed at least seven major lineages in the section *Calochroi *(Figure 3). The monophyly of *Calochroi*, *Caroviolacei*, *Dibaphi*, *Elegantiores*, *Napi*, *Pseudoglaucopodes *and *Splendentes *was supported in our maximum likelihood sequence analysis. ML and MP bootstrap support for *Rufoolivacei *and *Sulfurini *and MP bootstrap support for the monophyly of *Napi *was below 50%. The clades *Dibaphi*, *Elegantiores, Napi*, and *Splendentes *formed a well-supported monophyletic group in our maximum likelihood analysis together with *C. aureocalceolatus *and *C. natalis*, which appeared as a sister group to the remaining taxa of section *Calochroi*. *Cortinarius citrinus, C. langeorum, C. osmophorus, C. rapaceotomentosus *and *C. suaveolens *had isolated positions within the section *Calochroi *in our maximum likelihood analysis. In the maximum parsimony bootstrap analysis, *C. rapaceotomentosus *was supported as a sister taxon to the *Calochroi *clade with 70%, and *C. natalis*, *C. splendens *and *C. atrovirens *formed a well-supported clade (95% MP bootstrap).

### Reconstruction of ancestral states

Maximum parsimony reconstruction of ancestral states on the tree with the highest likelihood found for dataset 3 suggests that the bright pigmentation of the basidiomes and an abruptly marginate to flattened stipe bulb base evolved independently several times (Figure 3). Our results indicate that an abruptly marginate to flattened stipe bulb base is an autapomorphy for the *Calochroi *clade. The acquisition of bright flesh pigments appears as a synapomorphy for *Rufoolivacei*, *Pseudoglaucopodes *and *Sulfurini *and probably evolved independently in *C. citrinus *and *C. fulvocitrinus *as well as in *Splendentes *and *Elegantiores*.

### Characterisation of macro- and microscopical features

**Figure 4:**
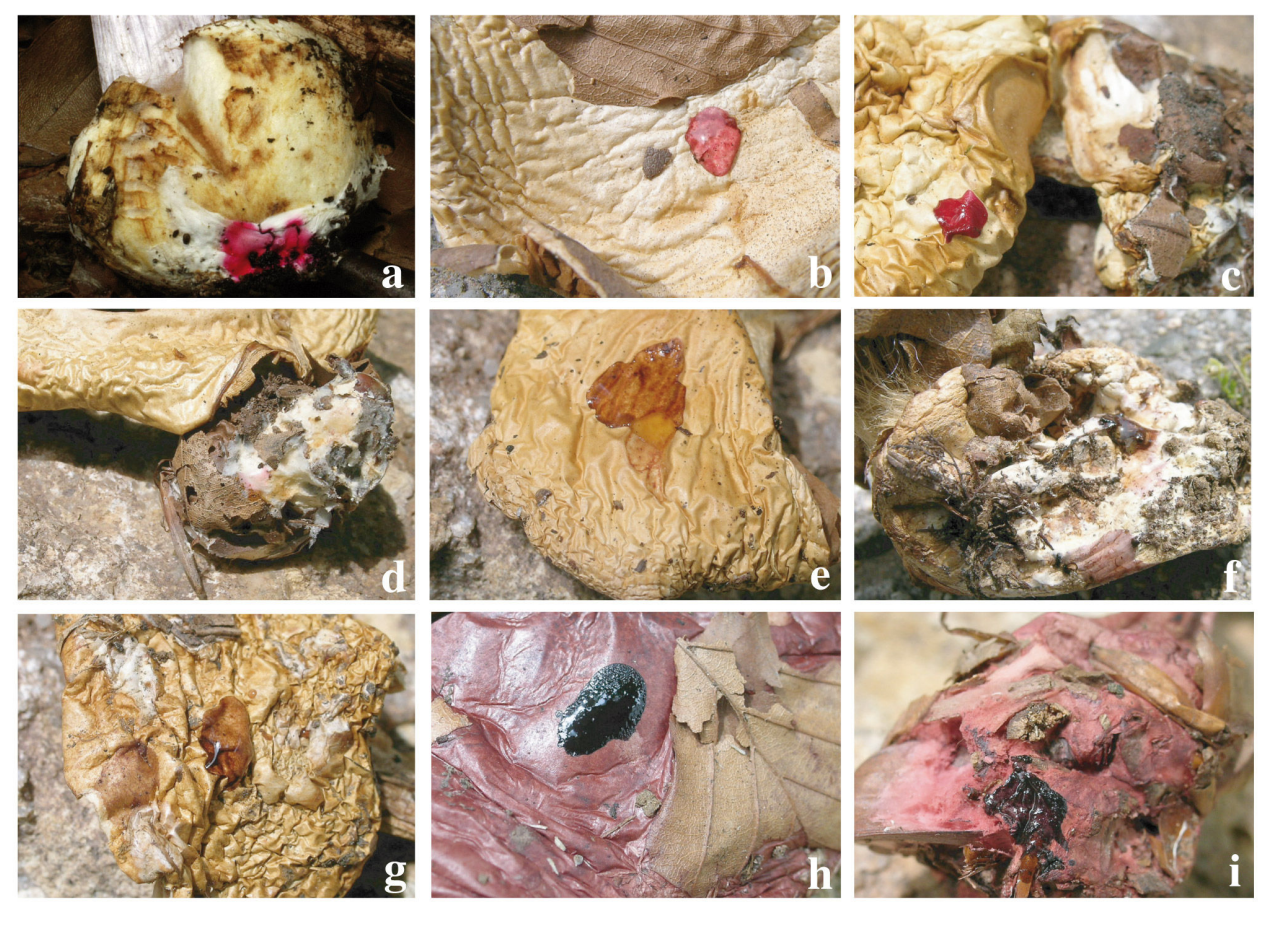
**Colour change reactions with 40% KOH on pileus surface and mycelia at the base of the stipe bulb in fresh and dried calochroid specimens**. **a**. Intense pink on mycelia (fresh material) in *C. insignibulbus *TUB 011657 (credit: G. Smith-Stohn). **b**. Pink on pileus surface in *C. sodagnitus *TUB 011428. **c**. Intense pink colour on pileus surface in *C. albertii *TUB 011850. d. Pink on mycelia in *C. sodagnitus *TUB 011428. **e**. Vinaceous on pileus surface in *C. suaveolens *TUB 011876. **f**. Vinaceous on mycelia in *C. eufulmineus *TUB 011876. **g**. Red-brown on pileus surface in *C. haasii *TUB 011858. **h**. Black on pileus surface in *C. rufoolivaceus *TUB 012739. **i**. Black on mycelia in *C. rufoolivaceus *TUB 012739.

**Figure 5:**
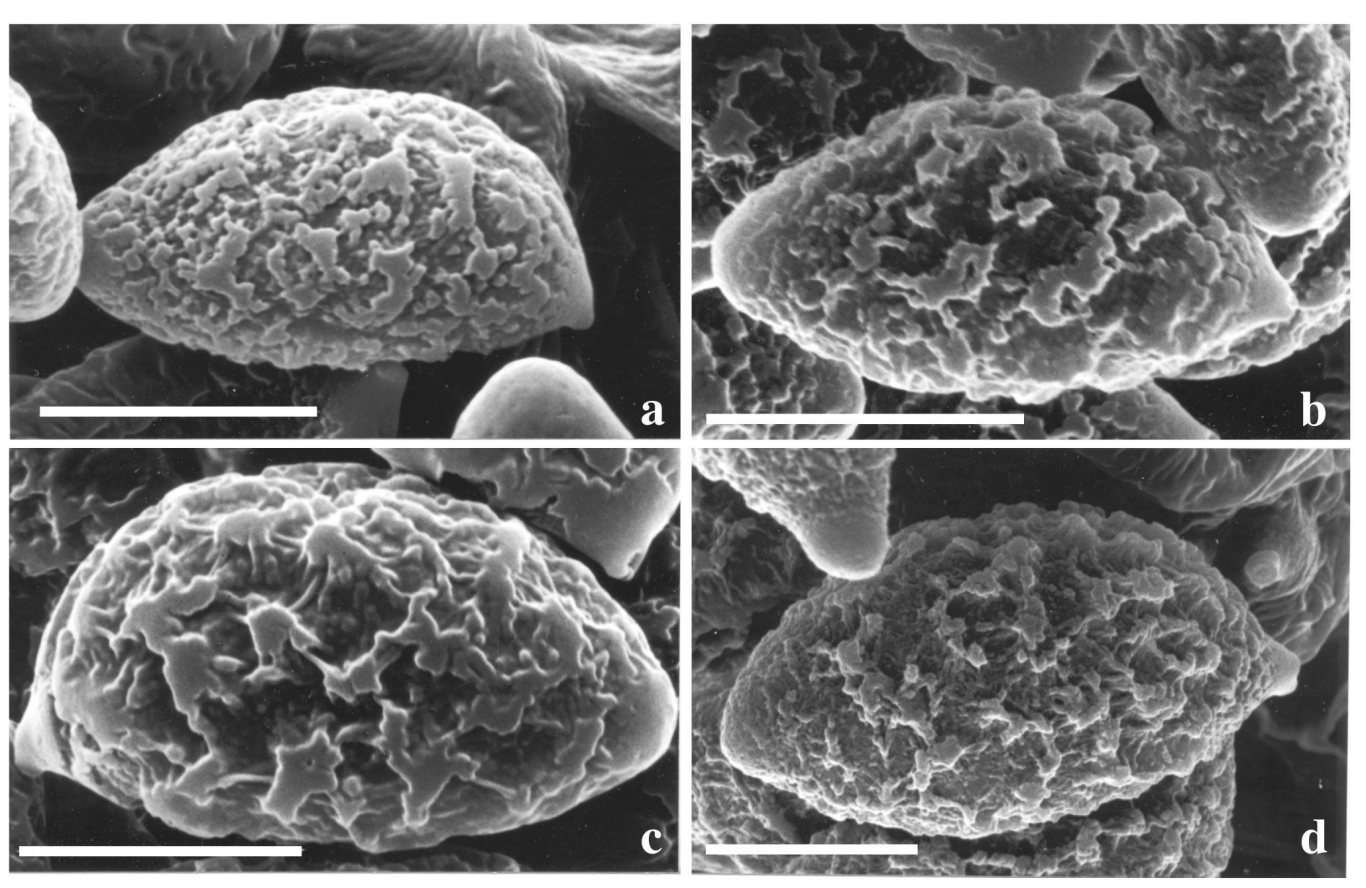
**Scanning electron micrographs of mature basidiospores of selected calochroid species**. **a**. *C. osmophorus *TUB 011399, **b**. *C. citrinus *TUB 011407, **c**. *C. rufoolivaceus *TUB 011405. **d**. *C. elegantior *TUB 011388. Note the presence of a distinctive papilla at the basidiospore apex. Bars = 4 μm.

Macroscopical features, including habit, basidiome coloration and veil coloration, macrochemical reactions with 40% KOH (see Figure 4), microscopical features of the pileipellis structure and hyphal coloration in 3% KOH, and basidiospore morphology (see Figure 5), are documented and compared for the *Calochroi *(see Additional file 2).

## Discussion

Although there have been previous molecular phylogenetic studies of *Calochroi *species [1,13,15,16] the present study is the first to include an extended, nearly complete sampling of the known species of this *Cortinarius *section across its natural range of distribution in Europe, Central America and North America (sampling does not include extensive areas of eastern North America). In general, our morphological and phylogenetic concepts for European calochroid species fit with those applied by Frøslev et al. [1,16] and Peintner et al. [12,14,26] (see Additional file 1). In the majority of cases, the evaluation of ranges in species variation is based on morphological and molecular data obtained from multiple collections and analysed with ITS sequences of 37 type specimens. These findings represent considerable taxonomic progress within a taxonomically complex group, and should make it much easier to either identify specimens or to name new species occurring across Europe, Central America and North America.

Phylogenetic analyses of the three final molecular datasets resulted in congruent and complementary trees (Figures 1, 2) and 3, but backbone resolution was higher in the trees inferred from the combined dataset 3 (Figure 3). Seven major lineages were supported within the section *Calochroi*, which occur throughout Europe, Central America and North America, and can be mostly circumscribed by a combination of features such as colour and colouration patterns, pigment content, colour reaction with KOH and similar shared indels in the RPB1 gene. In the following discussions, we focus on the most significant features circumscribing the major clades, and their degree of interspecific variation, as well as on specific geographical patterns of distribution. For each of the lineages discussed below, we give the branch support (ML/MP bootstrap values) obtained in the phylogenetic analyses of the combined dataset 3 (see Figure 3).

### *Calochroi *clade (99/98)

This clade is a species-rich lineage within the section *Calochroi *containing over thirty European and ten North American taxa (Figures 1 and 2). A combination of chemical and morphological features seems to support the monophyly of this clade, including similar colouration patterns of the context, stipe and pileus, KOH reaction, stipe shape and ecological requirements, such as edaphic and host specialisations as well as a shared 8 bp length indel in the intron 2 portion of the RPB1 gene (Figure 3). The indel is also present in two species (in *C. elegantissimus *and *C. rapaceotomentosus*) that were placed close to this lineage, but without significant support. Therefore, it is plausible that this indel represents an autapomorphy for an extended *Calochroi *clade. Within *Calochroi *as currently defined, there are some spectacular examples of similar basidiomes among distantly related species, which may be explained by similar evolutionary scenarios or by shared plesiomorphies. Thus, morphological features alone are often found to be confusing in delimiting and/or separating species, whereas DNA sequence analysis reveals the existence of a major number of still undescribed species that were overlooked in studies based solely on morphology. However, additional collections should be sequenced and thoroughly studied with respect to the degree of intraspecific variation of macroscopical, microscopical, chemical and ecological features before a formal description of any new species is completed. The fact that poor resolution within the *Calochroi *clade is a feature of both morphological and molecular analyses suggests that this lineage may have differentiated in a short period of time. Therefore, additional molecular markers may be needed to provide sufficient resolution within this lineage. Most species are rare, with limited distributions within boreo-nemoral areas, mainly growing on calcareous soils and associated with frondose trees. A number of species in this clade are strictly associated with frondose trees belonging to the Betulaceae (*Corylus*, *Carpinus*) and Fagaceae (*Castanea*, *Fagus*, *Quercus*), whereas relatively few species are known to be associated with coniferous trees of the Pinaceae (*Abies*, *Picea*, *Pinus*, *Pseudotsuga*) (see Figures 1 and 2).

### *Caroviolacei *clade (100/100)

This clade includes the European species *C. caroviolaceus*, *C. saporatus *and *C. rapaceoides *with no close relatives known to date in North America. Phylogenetic results from ITS + RPB1 + RPB2 sequences obtained by Frøslev et al. [1] placed these species in a weakly supported *Rufoolivacei *clade (see discussion of the *Rufoolivacei *clade). Moser [19] placed *C. caroviolaceus *and *C. saporatus *in separate stirpes within the subsection *Multiformes*. Members of this lineage are characterised by relatively large basidiomes with a distinctly rounded stipe bulb, pale context, amygdaliform basidiospores and a lack of colouration change with the application of KOH. The taxon *C. caroviolaceus *is exclusively associated with frondose trees belonging to the genera *Carpinus*, *Fagus *and *Quercus*, and *C. saporatus *occurs with *Fagus*, *Quercus *and *Tilia *in Europe.

### *Dibaphi *clade (99/98)

This clade contains European, Central American and North American populations of *C. arcuatorum *and the European species *C. dibaphus*. Members of the *Dibaphi *clade share similar colour changes with KOH, pigment content and colour patterns of the basidiomes. Chromogenic triterpenoid pigments (sodagnitins) in *C. arcuatorum *[10] and probably in other *Dibaphi *may be significant chemical markers for this group. Sequences of New World populations of *C. arcuatorum *differ significantly from their European counterparts, and they cluster into three closely related monophyletic groups, which roughly correspond to their respective geographical ranges of distribution. Despite relatively high sequence divergences between New World and European populations of *C. arcuatorum*, little phenotypic variation in morphological features of the basidiomes and colouration between these collections was observed. A certain range of variability of the basidiospore size was observed between collections of *C. arcuatorum *from different parts of North America and Europe [23]. However, the area sampled for this study represents only part of the modern pattern of geographical distribution of this species complex. Here, it is necessary to mention that an ITS sequence (AY033120; published by Peintner et al. [14]) of *C. arcuatorum *from Wyoming was identical to sequences of our European collections of this taxon. Populations of *C. arcuatorum *are associated with tree species of the genera *Fagus *and *Quercus *in Europe, *Quercus *spp., in Central America, and *Lithocarpus*, *Quercus*, *Pseudotsuga*, and *Tsuga *in North America. Our study suggests that some populations of the *C. arcuatorum *complex have evolved very specialised habitat requirements with allopatric diversification, probably involving co-migration along a cline with their associated host trees, specifically with Douglas fir, hemlock, stone oak and oak. Future studies may show that such populations already represent separated species. *Cortinarius dibaphus *seems to be less host specific growing with coniferous (*Abies alba*) and frondose (*Fagus sylvatica*, *Quercus *spp.) trees in Europe [2].

### *Elegantiores *clade (99/71)

This clade includes European (*C. elegantior*, *C. eufulmineus*, *C. glaucescens*, *C. majusculus*, *C. murellensis*, *C. olearioides*, *C. parafulmineus*, *C. splendificus*) and North American (*C. elegantior *JFA 11452 and JFA 11693, *C. elegantior *var.*americanus *JFA 12438) taxa. A phylogenetic analysis from ITS sequences published recently by Garnica et al. [25] revealed three subclades among European *Elegantiores*. Members of the *Elegantiores *clade share similar colouration of the pileus, lamellae, stipe and context, and KOH reaction. The yellow-greenish colours of the basidiomes of *C. eufulmineus*, *C. elegantior*, *C. olearioides *and *C. splendificus *correspond to flavomannin-di- and trimethylether pigments [7,9,11]. Populations from both sides of the distribution range of *C. elegantior *share remarkable morphological and colouration similarities. However, high levels of genetic diversity were found in the North American populations of *C. elegantior *as evidenced by various unique substitutions and indels (see Figures 1 and 2). Our phylogenetic analysis indicates that *C. elegantior *var.*americanus *represents a species separated from *C. elegantior *rather than a variety of it, which macroscopically differs mainly in the colour of the veil [18]. The genetic diversity found in the *C. elegantior *complex might suggest that populations evolved with their associated host trees. *Cortinarius elegantior *and *C. parafulmineus *are restricted to coniferous trees (*Picea, Pinus, Tsuga*) and the remaining taxa are associated with frondose trees of the genera *Carpinus*, *Fagus *and *Quercus *occurring across the boreo-nemoral areas in North America and Europe.

### *Napi *clade (85/<50)

This clade as conceived here contains European (*C. aureopulverulentus *and *C. napus*) and Northern American (*C. albobrunnoides, C. albobrunnoides *var. *violaceovelatus*, *C. aurora*, and *C. subpurpureophyllus *var. *sulphureovelatus*) taxa. Members in this clade have been classified in separate sections in the subgenus *Phlegmacium*: *C. aurora *was placed in the section *Aurantiovelati *[18]; *C. aureopulverulentus *in the section *Caerulescentes *[4]; *C. albobrunnoides*, *C. albobrunnoides *var. *violaceovelatus *and *C. napus *in the section *Phlegmacium *[see, for example [4,18,26]], and *C. subpurpureophyllus *var. *sulphureovelatus *in the section *Calochroi *[17]. The groupings derived from our molecular analyses are largely in agreement with similar colouration patterns of the pileus, lamellae, context and stipe, a lack of KOH reaction (except for *C. aurora *and *C. aureopulverulentus*) and basidiospore morphology in these taxa. The close placement of *C. aurora *and *C. aureopulverulentus *in our analysis (Figure 3) fits with similarities of the veil colouration. Moser and Peintner [26] analysed the phylogenetic relationships in the *Cortinarius aureopulverulentus *group. These authors demonstrated the paraphyly of the section *Thalliophili *[5], but the precise phylogenetic placement of *C. aureopulverulentus *was not resolved with confidence. On the basis of differences in veil colouration, Moser and Ammirati [17,27] distinguished the varieties *C. albobrunnoides *var. *violaceovelatus *and *C. subpurpureophyllus *var. *sulphureovelatus*. However, low sequence divergences (0.1%) among the European *C. napus *and the North American *C. albobrunnoides*, *C. albobrunnoides *var. *violaceovelatus *and *C. subpurpureophyllus *var. *sulphureovelatus *suggest that these taxa are conspecific or perhaps the ITS region is too conservative to discriminate among these species. The first alternative could suggest a surprising intraspecific lability of veil colouration. However, to clarify aspects on species delimitations, the analysis of additional fungal collections and DNA markers is necessary. Members of the *Napi *clade seem to have diversified together with coniferous trees of the genera *Abies*, *Picea*, *Pinus*, *Pseudotsuga *and *Tsuga *throughout boreo-nemoral areas in North America and Europe.

### *Pseudoglaucopodes *clade (100/100)

This lineage comprises European (*C. elotus, C. olivellus, C. pseudoglaucopus*) and North American (*Cortinarius *sp. USA 14 and 15, *C. elotoides, C. flavaurora*) taxa, congruent with current classification systems [see [4,18,19]]. Members of this clade share similar colouration patterns of the basidiomes, basidiospore morphology and lack of KOH reaction. This study indicates the need to re-evaluate the specific status of *C. elotoides *and *C. flavaurora *(Figures 1 and 2). Members of the *Pseudoglaucopodes *clade seem to have co-evolved strictly with coniferous trees of the genera *Picea *and *Pseudotsuga*, and these associations are maintained through subalpine regions in the northern hemisphere (North America and Europe).

### *Rufoolivacei *(<50/<50)

According to the present study, the monophyly of *Rufoolivacei *is questionable. This taxon includes the European species *C. cedretorum *var. *suberetorum*, *C. cupreorufus*, *C. elegantissimus*, *C. prasinus*, *C. odorifer*, *C. olivascentium*, *C. rufoolivaceus*, *C. xanthophyllus*, the North American collections *Cortinarius *sp. USA 7, USA 9, USA 10 and USA 12, and *C. saxamontanus*. Species in this group display several similarities including pigment content, KOH reaction, and colour patterns of the basidiomes. Oertel [9] and Steglich and Oertel [11] detected pigments of the phlegmacin group in the basidiomes of *C. elegantissimus*, *C. cedretorum*, and *C. prasinus *and of the rufoolivacin group in *C. cupreorufus*, *C. odorifer*, *C. rufoolivaceus*, and *C. xanthophyllus*, respectively. Interestingly, in the analysis of the combined dataset a cluster composed of European (*C. cedretorum *var. *suberetorum*, *C. cupreorufus*, *C. prasinus*, *C. rufoolivaceus*, *C. xanthophyllus*) and North American (*C. saxamontanus*) species received an ML bootstrap support of 86% (Figure 3). *Rufoolivacei*, as conceived here, differs substantially from the results of Frøslev et al. [1,16]. Our present study suggests that *Caroviolacei *plus *Pseudoglaucopodes *may represent a group distinct from *Rufoolivacei *(Figures 1 and 2). A combination of traits (colour of the basidiomes and KOH reaction) supports this hypothesis. Most Rufoolivacei are associated with frondose trees (*Fagus*, *Lithocarpus*, *Quercus*) and only few species occur with coniferous trees (*Picea*, *Pinus*, *Pseudotsuga*) across the boreo-nemoral regions in North America and Europe.

### *Splendentes *clade (77/88)

*Splendentes *includes relatively common European species (*C. atrovirens *f.*atrovirens*, *C. atrovirens *f.*ionochlorus*, *C. meinhardii*, *C. odoratus*, *C. splendens*) and also *C. viridirubescens *from North America. The phylogenetic placement of *C. viridirubescens *is consistent with the traditionally noted morphology, KOH reaction and colouration of the basidiomes [23]. This monophyletic group is in good agreement with similarities in pigment content, KOH reaction and colouration patterns of the basidiomes [15]. Ecologically, *C. atrovirens *f.*ionochlorus*, *C. odoratus *and *C. splendens *grow strictly associated with *Fagus sylvatica*; *C. meinhardii *with *Picea abies*; whereas *C. atrovirens *f.*atrovirens *and *C. viridirubescens *are less specific, growing with both frondose (*Arbutus*, *Fagus*, *Quercus*, *Lithocarpus*) and/or coniferous (*Abies*, *Picea*, *Pinus*, *Pseudotsuga*) trees.

### Sulfurini group (<50/<50)

Concerning *Sulfurini*, which includes European (*C. flavovirens *and *C. sulfurinus*) and North American (*C. guttatus*) species, our phylogenetic hypotheses are incongruent. On the one hand, *C. flavovirens *and *C. sulfurinus *appear as sister taxa in the best tree found in the ML analysis of the combined dataset 3 (though with bootstrap support below 50%; Figure 3); on the other hand *C. flavovirens *was separated from the joined *C. sulfurinus *and *C. guttatus *with a bootstrap support of 92% in the analysis of dataset 2 (Figures 1 and 2). Frøslev et al. [1] recognised *Sulfurini *(excluding *C. flavovirens*); as a monophyletic group. Members of the *Sulfurini *group share several similarities such as KOH reaction, pigment contents and colouration patterns of the basidiomes. The yellow-greenish colours of the basidiomes correspond to phlegmacin-8'-methylether B_2 _(*C. flavovirens *and *C. sulfurinus*) pigments [9,11]. *Cortinarius flavovirens *grows associated with frondose trees (*Fagus*, *Quercus*), and North American *C. guttatus *and *C. sulfurinus *are associated with coniferous trees (*Abies*, *Picea*, *Pinus*) in Europe.

### Species with unresolved phylogenetic positions

The isolated positions of *C. aureocalceolatus*, *C. aureofulvus*, *C. citrinus*, *C. langeorum*, *C. natalis*, *C. osmophorus*, *C. rapaceotomentosus *and *C. suaveolens *obtained in our maximum likelihood analysis are in part consistent with previous findings by Frøslev et al. [1,16]. *Cortinarius langeorum *and *C. suaveolens*, which were nested within the *Rufoolivacei *clade in the molecular analyses by these authors, were not included in *Rufoolivacei*, but appeared on isolated branches close to this group in our trees (Figures 1, 2 and 3). A close relationship between *C. rapaceotomentosus *and the *Calochroi *clade was supported in our MP bootstrap analysis. This hypothesis is consistent with a similar shared indel in the RPB1 sequences (Figure 3). The high MP bootstrap support for a position of *C. natalis *close to *Splendentes *is consistent with similar pigments of the basidiomes. *Cortinarius aureofulvus *has a wide distribution in subalpine forests across the northern hemisphere, where North American populations grow associated with *Abies lasiocarpa*, *Picea engelmannii*, *P. pungens *and *Pseudotsuga menziesii *[18], while their European counterparts occur associated with *Abies alba*, *Picea abies *and *Pinus sylvestris*.

### Multiple origins of sequestrate species in the section *Calochroi*

Thiers [28] and Thiers & Smith [29] suggested that the sequestrate forms in the genus *Cortinarius *represent morphological adaptations to dry environmental conditions. Molecular analyses indicated that sequestrate basidiomes forms have evolved independently several times within several clades in *Cortinarius *[12]. The same was concluded from the geographical distribution of modern populations and glacial pluvial events in the Great Basin of North America [30]. Sequestrate species appear during spring in subalpine to alpine sites across the western USA [29,30]. Our phylogenetic analyses suggest that sequestrate forms have evolved independently at least twice in the *Calochroi *clade (*C. verrucisporus *and *Cortinarius *sp. USA 16) and one time within *Rufoolivacei *(*C. saxamontanus*). Furthermore, this study emphasises the need to study the morphological and molecular data of additional specimens to clarify species concepts in these heavy veiled taxa.

### Circumscribing the major lineages within section Calochroi

Our concept of section *Calochroi *comprises species that have been classified in the sections *Calochroi *and *Fulvi *[2,31]; in the sections *Coerulescentes*, *Fulgentes*, *Glaucopodes*, *Laeticolores*, *Multiformes *and *Thalliophili *[5,32]; and in the sections *Calochroi*, *Coerulescentes*, *Fulvi *and *Laeticolores *[4], or *Calochroi*, *Coerulescentes*, *Fulvi *and *Scauri *[19]. Our phylogenetic results differ from those of Frøslev et al. [1,16] mainly regarding the number and the inner topology of the major clades recognised within the section *Calochroi*: (i) Frøslev et al. [1,16] recognised three (/C*alochroid*, /F*ulvi *and /*Rufoolivacei*) and five (/*Arcuatorum*, /*Calochroid*, /*Fulvi*, /*Rufoolivacei *and /*Sulphurinus*) major subclades within *Calochroi*, respectively; (ii) the members of the clades *Dibaphi*, *Elegantiores *and *Splendentes *in the present study were nested within the subclades /*Fulvi *and /*Rufoolivacei *of Frøslev et al. [16] and /*Arcuatorum*, /*Fulvi *and /*Rufoolivacei *of Frøslev et al. [1]; (iii) *Caroviolacei *and *Pseudoglaucopodes *were placed within a relatively weakly supported subclade assigned as /*Rufoolivacei *by Frøslev et al. [1,16]; (iv) the members of *Splendentes *were placed in isolated positions in the analyses of Frøslev et al. [16]. Some relationships among the *Dibaphi*, *Elegantiores*, *Napi *and *Splendentes *clades are clearly resolved for the first time in our combined analysis (Figure 3). Including additional molecular markers in future phylogenetic analyses may be the key to resolving more basal nodes in the *Calochroi *phylogeny.

Many species in the section *Calochroi *are characterised by bright colours on the one hand and by abruptly marginate to flattened bulbous stipe bases (see Figure 3). Most of the species in this clade are associated with broadleaf trees and generally grow under a layer of fallen leaves. Thus they are shielded from sunlight, whereas the presence of a broader stipe base allows more stability of larger basidiomes, especially in sites with a relatively thin layer of organic material. In our phylogenetic hypothesis obtained from the combined dataset (Figure 3) the species with brightly coloured pigments distributed throughout the whole basidiome and usually with rounded to marginate bulbous stipe base were placed in the basal parts of the tree, while members of the *Calochroi *clade and closely related species are characterised by their relatively pale coloured basidiomes (predominantly whitish to cream flesh and sometimes with greyish to violet tinges restricted above the lamellae, stipe apex and/or cortex) and abruptly marginate to flattened bulbous stipe base. Interestingly, there is striking homoplasy and phenotypic plasticity of basidiome colouration with age in species of the clades *Calochroi*, *Napi *and *Pseudoglaucopodes*. In contrast, the basidiomes of species of *Rufoolivacei *and the *Sulfurini *and *Splendentes *clades exhibit relatively little colour change with age. A possible explanation for this may be that the more highly methylated pigments of the latter group are more stable in relation to environmental factors (e.g. sunlight).

Our analyses show that colour reaction with KOH and colour of the basidiome context are the most phylogenetically informative characters to circumscribe major clades within the section *Calochroi *with only a few exceptions, whereas characters related to size and shape of the basidiomes, stipe shape, basidiospore size and shape, and pileipellis structure show high levels of homoplasy and/or phenotypic plasticity. Furthermore, characteristic colour reactions with KOH related to the presence/absence of sodagnitins either on the pileus and/or mycelium covering the stipe bulb offer a framework for discriminating among species with similar macroscopical appearance and ecological specialisation. This study demonstrates that dried and adequately preserved herbarium specimens are also useful for performing macrochemical tests with KOH. Similar observations were made in *Dermocybe *species on herbarium specimens stored for several years [33].

### Patterns of species diversification and biogeographic distributions

ITS DNA sequence divergence among several collections of species restricted to Europe, North America or Central America was low (0–0.1%). In general, our morphological and phylogenetic concepts for European calochroid species fit with those applied by Frøslev et al. [1,16] and Peintner et al. [12,14,27] (Additional file 1). Similar sequence variation was observed between collections from disjunct populations in the clade *Napi *(*C. napus/C. albobrunnoides/C. albobrunnoides *var. *violaceovelatus*/*C. subpurpureophyllus *var. *sulphureovelatus*) and *C. aureofulvus*, where sequence divergence was poorly related to geographical distances. For these examples, the ITS sequence divergence between North American and European populations is relatively low compared with the range of variation that has been detected in some biological species of Agaricales [34-37]. A possible explanation for the very low genetic variation between the populations mentioned above is that they were established through recent founder events and have not had time to evolve a higher ITS sequence divergence. Although long-distance dispersal explains well a conspicuous percentage of today's trans-Atlantic disjunct distributions in angiosperms (see [38] for a recent review), it seems unlikely that long-distance dispersal through basidiospores between continents is the reason for the low sequence divergence between North American and European populations of the taxa mentioned above, since a combination of several factors such as the presence of a compatible haploid mycelium, appropriated edaphic and climatic conditions, as well as the availability of adequate host tree(s) is necessary for successful colonisation and the development of basidiomes in the new habitat. Higher levels of sequence divergence, on the other hand, may indicate that populations have evolved faster or/and had a more ancient separation. Substantial sequence divergences have occurred for example within the lineages *Calochroi*, *Dibaphi *and *Elegantiores*, resulting in well-resolved phylogenetic relationships within these clades. Within the lineages *Dibaphi *and *Elegantiores*, we found that intracontinental relationships of European and North American populations were closer than the relationships between populations from distinct continents. Similar levels of genetic divergence were found in widely distributed species within the genus *Amanita *[39]. However, species/populations within the *C. arcuatorum *and *C. elegantior *complexes have undergone very little morphological changes since their divergence, making it impossible to separate them on the basis of morphological characters alone. Selection in these taxa may have predominantly occurred at the physiological level rather than at the morphological level. It is evident from these results that future studies including more representative population samplings are needed to clarify questions concerning distributional patterns and ranges of genetic divergence.

Host tree distributions seem to constitute one of the driving forces in the evolution of the genus *Cortinarius*. Main geographical distributions of species/populations in the section *Calochroi *include regional radiations (*Elegantiores *clade), as well as clinal (*Dibaphi*), boreo-nemoral (*Calochroi*, *Pseudoglaucopodes*) or intercontinental (*Napi, C. aureofulvus*) patterns throughout Europe, Central and North America. However, to understand modern geographical distributions in the section *Calochroi *better, it is necessary to consider the biogeographical history of their host trees [40-42] as well as geological events [see, for example, [43,44]]. Although the history of the flora is still somewhat controversial, generally it is accepted that migration either via the Bering or North Atlantic bridges during the late Cretaceous and the Tertiary periods has played a crucial role in modern distributions of plants [45]. Therefore, it is reasonable to assume that closely related *Cortinarius *species occurring on separate continents originated in the migration of a common ancestor together with its associated tree host.

The high diversification found in the *Calochroi *clade roughly matches the distribution of several broadleaf trees throughout boreo-nemoral areas [40] in Europe, Central and North America. An explanation for this high diversification may be that the edaphic factors (e.g., pH, soil aeration, organic and inorganic composition) of broadleaf forests are more favourable for many calochroid species than the coniferous forests, or simply the fact that there is a higher species diversity in the angiosperm trees compared to the conifers. The diversification of angiosperms and gymnosperms is thought to have driven the diversification of other ectomycorrhizal fungal groups [see e.g., [46-48]] and the *Calochroi *diversification might follow similar patterns. Despite their crucial role for the ecology of a wide spectrum of trees in the northern hemisphere (ectomycorrhiza), we are only beginning to understand the factors modelling the evolutionary history of the *Calochroi*. Future studies with a focus on molecular dating and phylogeographic analyses including a broad sampling of taxa occurring disjunctly in the northern hemisphere are required to estimate the age and geographical origins of the calochroid lineages as well as to further elucidate the historical dispersal and diversification.

## Conclusion

This study demonstrates the great potential of comparative DNA sequence analyses of multiple populations from different geographical origins for understanding patterns of genetic and morphological variation at the species/population level within a biogeographical and taxonomic context. Our phylogenetic analyses support the division of the section *Calochroi *into seven major clades (*Calochroi*, *Caroviolacei*, *Dibaphi*, *Elegantiores*, *Napi*, *Pseudoglaucopodes*, and *Splendentes*), whereas *Rufoolivacei *and *Sulfurini *were not supported as monophyletic entities. Major phylogenetic groups agree partially with morphology-based and earlier molecular phylogenetic studies. Some phylogenetic relationships within these clades are unexpected based on phenotypic features, which may be explained by convergent evolution of similar morphologies within phylogenetically divergent lineages in *Calochroi*. This study shows that combinations of features such as colour and colouration patterns, colour reactions with KOH, pigment contents, veil, and pileipellis colouration of basidiomes are useful for circumscribing clades, whereas characters related to size and shape of the basidiomes, stipe shape, basidiospore size and shape, and pileipellis structure display high levels of homoplasy and/or phenotypic plasticity. Bright pigments evolved independently multiple times in the section *Calochroi*, and abruptly marginate to flattened stipe bulbs represents an autapomorphy for the clade *Calochroi*.

Furthermore, our study suggests the need for future phylogenetic analyses using multiple molecular markers of those lineages with poorly understood phylogenies as well as the critical re-evaluation of species concepts especially within the clades *Calochroi*, *Dibaphi*, *Elegantiores*, *Napi *and *Pseudoglaucopodes*. Main geographical distributional patterns for *Calochroi *species include clinal, regional and disjunct intercontinental distributions across Europe, Central and North America, where species/populations seem to follow the natural distribution of their host trees. Species in the section *Calochroi *show different degrees of host fidelity: some are associated exclusively either with coniferous or frondose trees; while others show relatively high host fidelity at least at the host genus level, suggesting potential co-evolutionary/co-migratory patterns.

## Methods

### Taxon sampling

For preliminary molecular analyses we used a taxonomically representative global sampling of collections of *Cortinarius*, subgenus *Phlegmacium*, as a phylogenetic framework to filter out calochroid species which do not belong to our focal group, the monophyletic section *Calochroi sensu *Frøslev et al. [1,16] and Garnica et al. [15]. In a second step, thorough analyses were performed to estimate phylogenetic relationships within our focal group. Our final taxon sampling included collections of all known species of *Calochroi *and *Fulvi *sensu M.M. Moser [4,19] and Brandrud [2] with the exception of *C. chlorophanus*, *C. inexpectatus*, *C. rhodophyllus*, *C. stephanopus *and *C. vaginatus*. In addition, to establish congruence and uniformity of species concepts, our own sequences were compared with ITS sequences of European calochroid species retrieved from GenBank  and UNITE  databases. In many cases, multiple collections per species from different geographical origins were used to analyse intraspecific morphological and genetic variation. In addition to the specimens collected and identified in fresh condition by the authors and collaborators, selected herbarium collections from Austria (IB), France (PC), Norway (O), Spain (Arangu-Cort) and USA (MICH) were examined. Host tree genera were assigned to the collections based on field observations. A specimen list including geographical origin, host tree(s), voucher number and GenBank  accession numbers of the *Calochroi *species included in this study is available in the Supplementary Table (see Additional file 3).

### Extraction of genomic DNA, PCR amplification, and sequencing

The total genomic DNA was extracted from dried lamella fragments using the DNAeasy Plant Mini Kit (Qiagen, Hilden, Germany), according to the manufacturer's instructions. The ITS region (including the gene coding for the 5.8 S ribosomal subunit) and the D1/D2 regions of the nucLSU were amplified with the primer combination ITS1F (5'-CTTGGTCATTTAGAGGAAGTAA-3', [49])/NL4 (5'-GGTCCGTGTTTCAAGACGG-3', [50]). Alternatively, DNA from older or poorly preserved specimens was amplified with the primer combinations ITS1F/ITS4 (5'-TCCTCCGCTTATTGATATGC-3', [51]) and 5.8SR (5'-TCGATGAAGAACGCAGCG-3')/LR3 (5'-CCGTGTTTCAAGACGGG-3') [52]. PCR amplifications of RPB1 domains A-C were made with the primer combination RPB1-A (5'-GARTGYCCDGGDCAYTTYGG-3') and RPB1-C (5'-CCNGCDATNTCRTTRTCCATRTA-3') [53]. In a few cases, weak amplifications of the RPB1 gene were used as a template for a second PCR with the same primer combination. Amplified PCR products were purified using the QIAquick protocol (Qiagen). Cycle sequencing was accomplished using the ABI PRISM Dye-Terminator Cycle Sequencing Kit version 3.1 (Applied Biosystems, Foster City, CA, USA). For sequencing of the RPB1 A-C gene, the forward primer RPB1-A sg (5'-YTSAARGCYGGTGAGT-3') and the reverse primer RPB1-B sg (5'-TCCGCRCCYTCYTTGG-3') were used [54]. Sequencing was performed with an ABI 3100 automated sequencer (Applied Biosystems). Forward and reverse sequences were assembled and edited using Sequencher version 4.1 (Gene Codes Corporation, Ann Arbor, MI, USA).

### Alignments

Initially, contiguous sequences including ITS1 and ITS2, 5.8S and approximately 600 bases of the 5' terminal domain (D1/D2 regions) of the nucLSU of a broad sampling of *Phlegmacium *species by Garnica et al. [[13,15], unpublished data] were automatically aligned in MAFFT v5.7 [55] and analysed by neighbor joining [56] in PAUP* 4.0b10 [57] using Kimura 2-parameter distances [58]. A final sequence sampling was obtained by successive pruning towards the focal fungal group of this study. Those sequences lacking either ITS1 or ITS2 regions were excluded from the final matrix. We also aligned the nucleotide sequences of ITS region from our final alignment with those downloaded from GenBank  and UNITE  databases following the methodology indicated above (dataset 1). In addition, to estimate genetic variation between collections of the same species and also between closely related species more accurately, we aligned and compared our own sequences with their respective chromatograms and also those sequences retrieved from the GenBank and UNITE databases using Sequencher. Intraspecific genetic differences were counted from pairwise alignments. Furthermore, percentages of internal transcribed spacer region (ITS1, ITS1, and 5.8S subunit) and D1/D2 region of the rDNA sequence divergence within and among closely related species were estimated. We then realigned a final set of 230 ITS, 5.8S and D1/D2 sequences using the FFT-NS-i strategy of MAFFT (dataset 2). Sixty-one RPB1 A-C sequences were aligned using the E-INS-i strategy of MAFFT v5.7 [54], with minor manual adjustments made in SeAl v2.11 [59]. This alignment was concatenated with aligned ITS-5.8S-D1/D2 sequences from the same specimens to obtain dataset 3. Leading or trailing gaps were coded as missing data. Gblocks Version 0.91b [60] was used for eliminating gapped and too divergent portions of the datasets 2 and 3 with a minimum block length of 4 positions and gaps allowed in any alignment position for no more than half of the sequences. The full alignments may be obtained from TreeBase . After exclusion of the sites indicated above, the alignments had a length of 1144 bp (dataset 2 with 234 sequences) and 2381 bp (dataset 3 with 61 sequences).

### Molecular phylogenetic analyses

To establish congruence and uniformity of species concepts for European calochroid species (dataset 1) and to estimate phylogenetic relationships (datasets 2 and 3), maximum-likelihood (ML) analysis was performed with RAxML version 7.0.0 [61]. Doing so, we followed a new approach implemented in the software, which integrates bootstrap analysis [62] and heuristic searches for an optimal tree. First, 1000 bootstrap replicates were run, starting from maximum parsimony trees and using the GTRCAT approximation. Then, every 5^th ^bootstrap tree was used as a starting point for thorough ML analysis, using the GTRCAT model for heuristic searches, and optimising branch lengths and likelihood values of the final trees using the GTR + Γ model of DNA substitution. Tree graphics were generated in PAUP* [57] (Macintosh Classic version). For dataset 3 we additionally performed a maximum parsimony (MP) bootstrap analysis in PAUP*, involving 1000 replicates and treating gaps as missing data. In each replicate, ten rounds of heuristic search were run, with starting trees obtained by successive addition of sequences in random order and using the TBR branch swapping algorithm (storing multiple trees, steepest descent option in effect). The outgroup taxa (*Cortinarius caerulescens *UL 98/88, *C. glaucopus *TUB 011414, *C. variicolor *TUB 011416 and *C. violaceus *TUB 011561) were chosen on the basis of previous phylogenetic studies within genus *Cortinarius *[14,15].

### Ancestral state reconstruction

Ancestral states were reconstructed for two characters related to pigmentation and morphology; flesh colour: pale (whitish to cream, sometimes with greyish to violet tinges are present above the lamellae, stipe cortex and/or in stipe apex)/bright (yellow, olive, yellow green); shape of the stipe base: rounded to marginate/abruptly marginate to flattened. For this we used unweighted Wagner parsimony as implemented in PAUP* according to the ACCTRAN and the DELTRAN strategies [63] on the tree with the highest likelihood found. Since the used outgroup species differ from members of section *Calochroi *in their pigments and have a different stipe morphology, these taxa were excluded for the reconstruction of ancestral states (grey branches). For *Cortinarius *sp. USA 16 no data about these characters are available. Since the outgroup species differ from members of section *Calochroi *in their pigments and have a different stipe morphology, these taxa were excluded for the reconstruction of ancestral states.

### Macrochemical tests and morphological analyses

Fresh basidiomes were documented using comprehensive macroscopical descriptions accompanied with colour photographs. Macrochemical reactions with 40% KOH were performed by placing a drop directly on the tissue (surface and flesh of the pileus and stipe, and the mycelium covering the base of stipe bulb) of fresh and dried specimens, observing immediate and longer (5–10 minutes) colour changes. Changes in colour were documented from dried specimens with a NIKON COOLPIX 5400 digital camera. Microscopical characters were observed from both fresh and dried specimens mounted in H_2_O or 3% KOH solution. Pileipellis portions of dried specimens were soaked in 3% KOH for a few minutes before sectioning. The pileipellis structure from expanded pilei at a position halfway to the centre was studied from radial (longitudinal) free-hand sections using a razor blade and observed with ×40 and ×100 (oil immersion) lenses. Hyphal colour was recorded in 3% KOH. Scanning electron microscopy (SEM) of the basidiospores of selected species were made from approx. 5 mm^2 ^lamellae fragments fixed on double adhesive tape and sputter-coated with gold-palladium. Samples were examined with a Cambridge Stereoscan 250 Mk2 scanning electron microscope.

## Authors' contributions

SG designed the study, generated the DNA sequences, carried out morphological analyses and wrote the manuscript; MW and SG performed the phylogenetic analyses; MW, JA, BO and FO revised several versions of this manuscript. All authors approved the final manuscript.

## Supplementary Material

Additional file 1 **RAxML ML phylogram from an alignment of 576 calochroid sequences of ITS-5.8S.** Support values are derived from 1000 bootstrap replicates. GenBank accession numbers: DQ08XXXX are from [16]; DQ32XXXX, DQ3508242 and DQ66XXXX are from [1]; AY17XXXX are from [13], AY66XXXX from [15], EFO1XXXX from [25]; AF50XXXX are from [26]; AF3XXXXX and AY03XXXX are from [12] and [14], respectively; AJ236067 is from [65]; EUXXXXXX are from this study. UNITE accession numbers are indicated as UDBXXXXXX. Note: short sequences lacking either ITS1 or ITS2 were excluded from the final analysis. Click here for file

Additional file 2 **Distribution of macro- and microscopical features among calochroid species.** Macro- and microscopical features referring to habit, basidiome and veil coloration, macrochemical reaction with 40% KOH, pileipellis structure, hyphal coloration in 3% KOH and spore morphology were documented. Click here for file

Additional file 3 **Specimens used in this study and their respective collection sites, host tree(s), herbarium numbers and GenBank accession numbers.** Herbarium abbreviations: Arangu-Cort = Herbarium Sociedad Micológica Aranguren, Spain; IB = Herbarium Innsbruck, Austria; JFA = J. F. Ammirati, MES = Matthew Smith; MTS = Michelle Seidl, ST = Steve Trudell, BW = Ben Wood, University of Washington Herbarium (WTU), USA; KS-CO = private herbarium of Karl Soop; O = Herbarium Oslo, Norway; TUB = Herbarium Tubingense, University of Tübingen, Germany; OSC = Oregon State University; S = Herbarium Stockholm, Sweden; SCR = Sierra Research Center (Matthew Smith), University of California Berkeley (UCB), USA. ^a^Sequence from [13], ^b^Sequence from [15], ^c^Sequence from [25]. Click here for file
