# Supplementary material for: Phylogenetic relationships in Cortinarius, section Calochroi, inferred from nuclear DNA sequences
Source: BMC Evol Biol. 2009 Jan 2;9:1. doi: 10.1186/1471-2148-9-1 (PMC2653478; doi:10.1186/1471-2148-9-1)
Supplement: Additional file 2 — Distribution of macro- and microscopical features among calochroid species. Macro- and microscopical features referring to habit, basidiome and veil coloration, macrochemical reaction with 40% KOH, pileipellis structure, hyphal coloration in 3% KOH and spore morphology were documented. [file 1471-2148-9-1-S2.doc]

**Characterisation of macroscopical features**

Habit

Most of the species in the section *Calochroi* were characterised by exposed basidiomes with a well-developed and bulbous (rounded to marginate or adruptly marginate to flattened) stipe base, and a fibrillose (cortinate) veil, whereas the North American species *Cortinarius* sp. USA 16, *C. saxamontanus*, and *C. verrucisporus* were characterised by partially hypogeous basidiomes with a poorly developed stipe, occasionally tapering slightly toward the base, and a heavy, long-lasting thick membranous veil, but retaining the production of ballistospores.

Basidiome coloration

A spectacular diversity of striking colours and colour patterns of basidiomes was found among the *Calochroi* species included in this study. There was a high degree of intraspecific variation in colouration, dependent on the age of the basidiomes and environmental factors, for example, exposed as compared to covered pilei within the same collection. Main colouration patterns circumscribing the major clades within the section *Calochroi* may be summarised as follows: (1) the *Calochroi* and *Caroviolacei* clades include species with a whitish, pale yellowish, yellow-citrine or yellow-ochraceous to brown-ochraceous pileus; white stipe and lamellae; often with violet-lilac tinges in the upper part of the stipe and sometimes also on the pileus surface; and a context colour that ranged from mostly whitish (yellow-green in *C. fulvocitrinus*) to sometimes violet (mainly in the stipe apex); (2) the *Dibaphi* clade comprises species with a pale lilac, grey-lilac or lilac brownish pileus; pale grey, lilac-grey to lilac-blue lamellae; a bright lilac or ochraceous to pale yellow-brownish stipe; and a colour context that ranged from mostly whitish to violet (mainly in the stipe apex); (3) members of the *Elegantiores* clade were usually characterised by yellowish, ochre-brown or red-brown colours mixed with an orange tinge on the pileus; yellow to ochraceous lamellae; a pale to bright yellow or yellow-ochraceous stipe; and a predominantly pale yellowish to bright yellow context; (4) the *Napi* clade contains species with pileus colours ranging from yellowish (on the margin) to frequently dark red-brown or lilac (*C. flavobulbus*); lilac, blue-violet or grey lamellae, stipes with a lilac-grey apex; whitish lower parts (the stipe is completely white in *C. napus*); and a context ranging from mostly white to sometimes violet in the pileus and stipe apex; (5) *Pseudoglaucopodes* includes species with a pale yellow, olive-brown, olive-ochraceous, ochraceous-brown or reddish-brown pileus; yellow, olive-yellow, greyish green or lilac (*C. elotoides*, *C. elotus*, *C. pseudoglaucopus*) lamellae; a white, yellow, pale yellow-green or violet (*C. elotus*, *C. pseudoglaucopus*) stipe; and a context that is mostly white (olive-green in *C. olivellus*), but sometimes violet in the pileus context and stipe apex; (6) *Rufoolivacei* includes species with a sulphur-yellow, olive-green or red-brown to red-purple pileus; yellow, olive, yellow-green to lilac (*Cortinarius* sp. USA 17) lamellae; a yellowish, olive, yellow-green or lilac (*Cortinarius* sp. USA 17, *C. cedretorum*, *C. rufoolivaceus*) stipe; and a mostly white to yellow-green and sometimes (in the stipe apex) lilac context; (7) members of the *Splendentes* clade were characterised by olive-green, bright yellow-green or dark-green to olive-brown colours on the pileus; yellow, sulphur yellow or greenish lamellae; a pale yellow, sulphurous or yellow-green stipe; and a bright yellowish-green context; (8) *Sulfurini* includes species with an olive-yellowish, yellow-green, olive-brown pileus; pale yellow, green-yellow or sulphur yellow lamellae; a pale yellow-green to greenish stipe; and a yellow-greenish context. Violet-lilac colours of the lamellae, surface and context of the stipe apex, pileus margin, and/or veil were found in representatives of the clades *Calochroi* (except *C. aurantiorufus*, *Cortinarius* sp. USA 13, *C. fulvocitrinus*, *C. ochraceopallescens*, *C. osmophorus*), *Caroviolacei* (except *C. saporatus*), *Dibaphi*, *Napi*, *Pseudoglaucopodes* (except *Cortinarius* sp. USA 14, *C. flavaurora*, *C. humolens*, *C. olivellus*), *Rufoolivacei* (except *Cortinarius* sp. USA 8, USA 9, and USA 12, *C. claroflavus*, *C. elegantissimus*, *C. guttatus*, *C. prasinus*), *Splendentes* (except *C. meinhardii*, *C. odoratus*, *C. splendens*), and in *C. aureocalceolatus* and *C. suaveolens*. Similar colouration patterns of the pileus, stipe and lamellae were observed among distantly related species within the clades *Calochroi*, *Dibaphi*, and *Elegantiores*.

Veil colouration

The examined *Calochroi* species showed a large intra- and interspecific variation in the coloration of the veil. In young basidiomes, the universal veil at the bulb margin was violet-lilac in some species belonging to the clades *Calochroi* (e.g., *C. cordata*, *C*. *lilacinovelatus*, *C. molochinus*, *C. sodagnitus, C. violaceipes*), *Dibaphi*, *Napi* (e.g., *C. flavobulbus*), *Pseudoglaucopodes* (*C. elotus*, *C. pseudoglaucopus*) and *Rufoolivacei* (e.g., *C. rufoolivaceus*); whitish in species mainly within the clades *Calochroi*, *Caroviolacei* and *Pseudoglaucopodes* (e.g. *C. claroflavus*, *C. flavaurora*); yellow, yellow-ochraceous to olive-green usually in the clades *Napi*, *Calochroi*, *Elegantiores*, *Rufoolivacei*, *Splendentes*, and *Sulfurini,* and in *C. aureofulvus*; and orange in *C. aurora* (*Napi* clade). Veil remnants forming more or less concentric and dark coloured scales toward the pileus margin characterised some members of the clades *Calochroi* (e.g., *C. fulvocitrinus*), *Elegantiores* (e.g. *C. majusculus*), *Splendentes*, and *Sulfurini*.

Macrochemical reaction

Several colour patterns were observed with the application of 40% KOH on the pileus and stipe surface, and the context of both fresh and dried basidiomes. The main colour patterns on the pileus surface and basal mycelia (underneath the stipe bulb) from dried specimens (Figure 3) may be summarised in five categories: (1) a distinctive pink reaction on pileus surface and basal mycelium was observed in taxa belonging to the clades *Calochroi* (*Cortinarius* sp. USA 4 and USA 13,*C. albertii*, *C. catharinae, C. cisticola*, *C. molochinus*, *C. nymphicolor*, *C. subgracilis*, *C. sublilacinopes*, *C. sodagnitus*)*, Dibaphi* (European collections of *C. arcuatorum*, *C. dibaphus*), *Napi* (*C. aureopulverulentus*), and *Rufoolivacei* (*C. flavobulbus*); (2) a vinaceous to red-brown or no reaction on the pileus surface, and a pink reaction on the basal mycelium was found in members of the clades *Calochroi* (*C. aurora*, *C. barbarorum*, *Cortinarius* sp*.* USA 1, USA 4, USA 5 and USA 6, *C. calochrous*, *C. cordata*, *C. elegantior* JFA 11693, *C. insignibulbus*, *C. laberiae*, *C. violaceipes*), *Dibaphi* (North American collections of *C. arcuatorum*), *Elegantiores* (*C. elegantior* JFA 11693), *Napi* (*C. albobrunnoides* var. *violaceovelatus*, *C. aurora*, *C. subpurpureophyllus* var. *sulphureovelatus*) and *Sulfurini*, and in *C. aureocalceolatus* and *C. langeorum*; (3) a vinaceous to red-brown reaction on the pileus surface and a vinaceous reaction on the basal mycelium in the *Elegantiores* clade (except *C. elegantior* JFA 11693), and in *C. natalis*, *C. olivascentium* and *C. suaveolens*; (4) a brown, brownish-pink or red-brown to dark red-brown reaction on the pileus surface and no colour reaction on the basal mycelium characterised representatives of the clades *Calochroi* (*Cortinarius* sp. EUR 1, USA 2 and USA 3, *C. aurantiorufus*, *C. corrosus*, *C. frondosophilus*, *C. haasii*, *C. largentii, C. lilacinovelatus, C. piceae*, *C. roseobulbus*, *C. vesterholtii*), *Pseudoglaucopodes* (except *Cortinarius* sp. USA 14 and USA 15, *C. elotoides*) and *Caroviolacei* (*C. rapaceotomentosus*); (5) a black reaction on the pileus surface and basal mycelium characterised some members of the clades *Calochroi* (*C. fulvocitrinus*), *Rufoolivacei*, *Splendentes*, and *C. aureofulvus*.

**Characterisation of microscopical features**

Pileipellis structure

The investigated calochroid species (except *C. aureocalceolatus)* were characterised by a *pileipellis simplex* composed of a usually thick epicutis of several strata of tightly connected and mostly pigmented hyphal elements, often with epiparietal encrustations (usually zebra-striped) or embedded in a coloured matrix, and a well-developed gelatinous layer of relatively thin, subradially to irregularly ascending hyphae, especially toward the pileus margin. The hyphae of the veil on the pileus surface were thin, colourless or, more commonly, with parietal or intracellular pigmentation, frequently with parietal incrustations, often becoming collapsed and filled with colored necropigment with age.

Hyphal colouration in 3% KOH

The hyphae of the epicutis, the gelatinous layer and velar remnants on the pileus surface showed characteristic colour reactions with 3% KOH. Hyphae of the epicutis and the gelatinous layer turned pink in members of the clades *Calochroi* (*C. albertii*, *C. barbarorum*, *C. catharinae, C. cisticola, C. molochinus, C. nymphicolor*, *C. subgracilis*, *C. sodagnitus*), *Dibaphi* and *Napi* (*C. aurora*); and vinaceous in the clades *Calochroi* (*C. caesiocinctus*, *C. cordata*, *C. fulvocitrinus*, *C. laberiae*, *C. ochraceopallescens*, *C. roseobulbus*, *C. sublilacinopes*, *C. verrucisporus*, *C. violaceipes*), *Napi* (*C. aureopulverulentus*), *Elegantiores*, *Rufoolivacei,* *Splendentes* and *Sulfurini,* and in *C. aureofulvus* and *C. natalis*. The veil hyphae on the pileus surface turned olive-green in species of the clades *Calochroi* (*C. violaceipes*), *Elegantiores* (*C. majusculus*), *Napi* (*C. aurora*), *Pseudoglaucopodes* (*Cortinarius* sp. EUR 2, *C. praetermissus*), *Rufoolivacei*, *Splendentes*, and *C. glaucescens*, and red-brown in members of the clades *Calochroi* (*Cortinarius* sp. EUR 1, *C. aurantiorufus*, *C. caesiocinctus, C. haasii*, *C. ochraceopallescens, C. subgracilis*, *C. selandicus*, *C. sublilacinopes*), *Elegantiores* (except *C. majusculus*), and *Caroviolacei* (*C. rapaceotomentosus*).

Basidiospore morphology

Basidiospore shape varied from amygdaliform or broadly ellipsoid to citriform or subcitriniform; the spores were moderately to coarsely verrucose, with isolated to interconnected warts, and often with a distinct suprahilar plage. The subcitriform to citriform basidiospores usually had a distinctive and often smooth apical papilla (Figure 4).
