# Supplementary material for: Phylogenetic relationships in Cortinarius, section Calochroi, inferred from nuclear DNA sequences
Source: BMC Evol Biol. 2009 Jan 2;9:1. doi: 10.1186/1471-2148-9-1 (PMC2653478; doi:10.1186/1471-2148-9-1)
Supplement: Additional file 3 — Specimens used in this study and their respective collection sites, host tree(s), herbarium numbers and GenBank accession numbers. Herbarium abbreviations: Arangu-Cort = Herbarium Sociedad Micológica Aranguren, Spain; IB = Herbarium Innsbruck, Austria; JFA = J. F. Ammirati, MES = Matthew Smith; MTS = Michelle Seidl, ST = Steve Trudell, BW = Ben Wood, University of Washington Herbarium (WTU), USA; KS-CO = private herbarium of Karl Soop; O = Herbarium Oslo, Norway; TUB = Herbarium Tubingense, University of Tübingen, Germany; OSC = Oregon State University; S = Herbarium Stockholm, Sweden; SCR = Sierra Research Center (Matthew Smith), University of California Berkeley (UCB), USA. aSequence from [13], bSequence from [15], cSequence from [25]. [file 1471-2148-9-1-S3.doc]

| Taxa | Host tree(s) and collection site | Herbarium No. | GenBank No.  ITS, 5.8S and D1/D2 rDNA | GenBank  No.  RPB1  A-C |
| --- | --- | --- | --- | --- |
| *C. albertii* Dima, Frøslev & T.S. Jeppensen | *Fagus sylvatica*, Ebringen (Schönberg), Germany | TUB 011850 | AY669560^b^ | EU088202 |
|  | *Fagus sylvatica*, Ebringen (Schönberg), Germany | TUB 012696 | EU056989 |  |
| *C. albobrunnoides* M.M. Moser & McKnight var. *violaceovelatus* M.M. Moser & Ammirati | *Picea engelmannii, Pseudotsuga menziesii* and *Abies lasiocarpa,* Washington: Chelan Co., Lake Ann, USA | JFA 10070  Isotype | EU057015 |  |
| *C. alnobetulae* Kühner ex Kühner [= *C. moseri* (E. Horak) E. Horak] | *Alnus viridis*, scattered *Picea abies* and *Larix decidua* trees, Passo del Rolle, Italy | JFA 12247 | EU655672 |  |
| *C*. *arcuatorum* Rob. Henry | *Fagus* *sylvatica*, *Pinus* sp*.*, and *Picea* *abies*, Tübingen, Germany | TUB 011421 | AY174822^a^ |  |
|  | *Fagus sylvatica*, Buir (Eifel), Germany | TUB 011447 | AY174823^a^ |  |
|  | *Fagus sylvatica* and *Quercus* sp., Eschweiler, Germany | TUB 011403 | AY174824^a^ | EU088228 |
|  | *Lithocarpus densiflora*, *Quercus chrysolepis*, *Umbellularia californica*, and *Pseudotsuga menziesii,* California: Del Norte Co., Patrick Creek Campground, USA | JFA 11765 | EU057005 |  |
|  | *Lithocarpus densiflora*, *Quercus chrysolepis*, *Umbellularia californica*, and *Pseudotsuga menziesii,* California: Del Norte Co., Patrick Creek Campground, USA | JFA 11766 | EU057004 |  |
|  | *Lithocarpus densiflora*, California: Del Norte Co., Big Flat Station. S. Fork Smith River, USA | JFA 11803 | EU057003 |  |
|  | *Quercus* *seemannii* and *Q. copeyensis*, Jardín de Dota, San Jose, Empalme, Costa Rica | JFA 12037 | EU057060 |  |
|  | *Quercus* *seemmannii* and *Q. copeyensis*, Jardín de Dota, San Jose, Empalme, Costa Rica | JFA 12039 | EU057000 |  |
|  | *Quercus costaricensis*, Prov. Cartago: Parque Prusia, Costa Rica | JFA 12061 | EU057001 |  |
|  | *Lithocarpus densiflora* and *Tsuga heterophylla*, California: Mendocino Co., Casper-Little Lake Road, USA | JFA 11893 | EU057002 |  |
| *C*. *atrovirens* Kalchbr. | *Picea abies* and *Abies alba*, Locherhof, Germany | UL 96/81 | AY174848^a^ |  |
|  | *Picea abies*, *Abies alba,* and *Fagus sylvatica*, Oberjoch, Germany | TUB 011639 | EU057011 | EU088233 |
| *C*. *atrovirens* Kalchbr. [*ionochlorus* morph] | *Quercus suber*, Caldana, Italy | TUB 012716 | EU057010 |  |
| *C*. *atrovirens* Kalchbr. [*ionochlorus* morph] | *Fagus sylvatica* and *Quercus* sp., Eschweiler, Germany | TUB 011430 | AY174834^a^ |  |
| *C. aurantiorufus* Bidaud | *Quercus faginea,* Villarcayo, Burgos, Spain | Arangu-Cort-03110802 | EU057058 |  |
|  | *Quercus suber,* Laiana, Prov. Grosseto/Toscana, Italy | TUB 012776 | EU057072 |  |
|  | *Quercus ilex*, Sant Feliu de Pallerols, Gerona, Spain | Arangu-Cort-03102002 | EU057056 |  |
|  | *Quercus ilex* and *Pinus halepensis,* Port-de-Bouc, France | TUB 011638 | EU056987 |  |
|  | *Fagus sylvatica* and *Quercus* sp., Eschweiler, Germany | TUB 011647 | EU655684 |  |
| *C*. *aureocalceolatus* M.M. Moser & Peintner | *Fagus sylvatica*, Ebringen (Schönberg), Germany | TUB 011842 | AY669569^b^ |  |
|  | *Fagus sylvatica*, Luziert, France | TUB 011640 | EU057013 |  |
|  | *Fagus sylvatica*, Pfaffenweiler, Germany | TUB 012685 | EU057014 | EU088229 |
| *C*. *aureofulvus* M.M. Moser | *Picea abies*, Wolterdingen, Germany | TUB 011831 | AY669571^b^ |  |
|  | *Abies lasiocarpus, Tsuga heterophylla, T. mertensiana, Pseudotsuga menziesii, Picea engelmannii*, and *Pinus monticola,* Washington: Skagit Co., Easy Pass Trailhead, USA | JFA 10065 | EU057051 |  |
|  | *Abies lasiocarpa, Picea engelmannii*, *Pinus contorta, P. flexilis*, *Salix* sp., and *Populus* *tremuloides*, Colorado: Boulder Co., Mountain Research Station, USA | JFA 12428 | EU057061 |  |
| *C*. *aureopulverulentus* M.M. Moser | *Picea abies*, Dunningen, Germany | TUB 011843 | AY669568^b^ |  |
| *C. aurora* M.M. Moser & Ammirati | *Picea engelmannii* and *Pinus contorta*, Wyoming: Teton Co., Fourmile Meadow, USA | JFA 9938  Isotype | EU057018 |  |
|  | *Picea engelmannii*, Oregon: Linn Co., Crescent Mountain Trail, USA | JFA 12724 | EU057019 | EU088231 |
| *C. barbaricus* (Brandrud) Frøslev, T.S. Jeppensen & Brandrud | *Picea abies* and *Pinus* sp., Hällekis, Sweden | S: F16320 | EU056978 |  |
| *C. barbarorum* Bidaud*,* Moënne-Locc. & Reumaux | *Pinus sylvestris* and *P. pinaster,* Valle de Tobalina, Burgos, Spain | Arangu-Cort-03103102 | EU057053 |  |
|  | *Picea abies*, Waldhausen, Kirnbergsee, Germany | TUB 011648 | EU655671 |  |
| *C*. *caesiocinctus* Kühner ex Kühner | *Picea abies*, Härjedalen, Sweden | KS-CO 1278 | EU056969 |  |
| *C*. *calochrous* (Pers.: Fr.) Fr. | *Fagus sylvatica,* Gerolstein-Gees, Germany | TUB 011398 | AY174838^a^ |  |
|  | *Fagus sylvatica*, Urft, Germany | TUB 011442 | AY174830^a^ |  |
|  | *Fagus sylvatica,* Valle de Salazar, Navarra, Spain | Arangu-Cort-03101301 | EU057066 |  |
|  | *Fagus sylvatica*, Tuttlingen-Esslingen, Germany | TUB 011643 | EU056983 |  |
|  | *Fagus sylvatica*, Sulz, Germany | TUB 011649 | EU660938 |  |
|  | *Fagus sylvatica*, Sulz, Largenhard, Germany | TUB 011650 | EU660940 |  |
|  | *Fagus sylvatica*, Loogh, Germany | TUB 011654 | EU684536 |  |
| *C*. *caroviolaceus* P.D. Orton | *Quercus* sp. and *Corylus avellana*, Ostrya, Italy | TUB 011849 | AY669559^b^ |  |
|  | *Quercus* *suber*, Laina, Italy | TUB 012692 | EU057049 |  |
|  | *Fagus sylvatica*, Eimersdorf, Germany | TUB 012693 | EU056943 | EU088221 |
|  | *Carpinus* *betula*, *Quercus* sp., and *Fagus sylvatica*, Karlstadt, Germany | TUB 012694 | EU056945 |  |
|  | *Quercus* sp*.,* Caldana, Italy | TUB 012695 | EU056944 |  |
| *C*. *catharinae* Consiglio | *Quercus ilex* and *Quercus faginea,* Burgos, Spain | Arangu-Cort-03102502 | EU057065 |  |
|  | *Fagus sylvatica*, Wöllmisse, Germany | TUB 011642 | EU056967 |  |
|  | *Fagus sylvatica* and *Quercus* sp*.*, Ebringen (Schönberg), Germany | TUB 012689 | EU056968 |  |
|  | *Fagus sylvatica*, Ebringen (Schönberg), Germany | TUB 012690 | EU056965 |  |
|  | *Fagus sylvatica*, Sulz-Ernet, Germany | TUB 011641 | EU056966 |  |
|  | *Fagus sylvatica*, *Carpinus* *betulus*, and *Quercus* sp., Heugrumbach, Germany | TUB 011644 | EU057073 | EU655644 |
| *C*. *cedretorum* Maire var. *cedretorum* | *Picea abies*, Singener Berg, Thuringia, Germany | TUB 011651 | EU655685 |  |
| *C*. *cedretorum* Maire var. *suberetorum* Maire | *Fagus sylvatica*, Lahr-Sulz, Germany | TUB 011851 | AY669564^b^ |  |
|  | *Quercus* *suber*, Caldana, Italy | TUB 012698 | EU057048 | EU088235 |
| *C. cisticola* Frøslev & T.S. Jeppensen | *Tilia* sp. and *Corylus avellana*, Munkkängarna, Sweden | S: F36828 | EU056957 |  |
|  | *Carpinus betulus*, Freyburg/Unstrut, Germany | TUB 011652 | EU655680 | EU655641 |
| *C*. *citrinus* J.E. Lange ex P.D. Orton | *Fagus sylvatica*, Gerolstein-Gees, Germany | TUB 011407 | AY174821^a^ | EU088211 |
|  | *Fagus sylvatica*, Wöllmisse, Germany | UL 99/87 | AY174820^a^ |  |
|  | *Fagus sylvatica*, Loogh (Eifel), Germany | TUB 011452 | AY174825^a^ |  |
| *C. cordata* Frøslev | *Quercus ilex* and *Quercus faginea,* Mena, Burgos, Spain | Arangu-Cort-00/24 | EU057064 |  |
|  | *Fagus sylvatica*, Bois d’Hufelz, Kitzing, France | TUB 011653 | EU655669 | EU655638 |
| *C*. *corrosus* Fr. | *Picea abies*, *Pinus* sp., and *Abies* *alba*, Kirnbergsee, Germany | TUB 012705 | EU056975 |  |
|  | *Picea abies*, *Pinus* sp. and *Abies* *alba*, Zindelstein, Germany | TUB 012706 | EU056974 | EU088206 |
|  | *Pinus sylvestris, Populus tremula,* and *Corylus avellana,* Martinet, Gerona, Spain | Arangu-Cort-01102201 | EU057057 |  |
| *C*. *cupreorufus* Brandrud | *Picea abies*, Tannheim, Austria | TUB 011418 | AY174831^a^ |  |
|  | *Picea abies*, Ammurbach, Germany | TUB 012707 | EU056992 |  |
| *C*. *dibaphus* Fr. | *Abies alba*, Pfaffenweiler, Germany | TUB 012708 | EU057006 |  |
|  | *Abies alba*, Levier, France | TUB 011437 | AY174819^a^ | EU088230 |
| *C*. *elegantior* (Fr.) Fr. var. *elegantior* | *Picea abies*, Oberjoch, Germany | TUB 011388 | AY174850^a^ | EU088225 |
|  | *Picea abies*, Oberjoch, Germany | TUB 011394 | AY174851^a^ |  |
|  | *Picea abies*, Tannheim, Austria | TUB 012709 | EF014262^c^ |  |
|  | *Tsuga heterophylla* and *Picea sitchensis*, Oregon: Lincoln Co., Drift Creek Camp, USA | JFA 11693 | EU056997 |  |
|  | *Picea engelmannii* and *Pinus contorta*, Wyoming: Teton Co., Flagstaff Road, USA | JFA 11452 | EU056998 |  |
| *C*. *elegantior* (Fr.) Fr. var. *americanus* M.M. Moser & McKnight | *Picea engelmannii* and *Pinus contorta*, Wyoming: Carbon Co., Snowy Range, below Silver Lake, USA | JFA 12438 | EU056999 |  |
| *C*. *elegantissimus* Rob. Henry ex Rob. Henry | *Fagus sylvatica*, Sulz-Ernet, Germany | TUB 012710 | EU057037 |  |
|  | *Fagus sylvatica*, Tuttlingen-Esslingen, Germany | TUB 011855 | AY669565^b^ | EU088214 |
| *C. elotoides* M.M. Moser & McKnight | *Picea engelmannii* and *Pinus contorta*, Wyoming: Teton Co., Fourmile Meadow, USA | JFA 9983 | EU056948 |  |
| *C*. *elotus* Fr. | *Picea obovata*, Uagan Unus, Russia | IB 2001/0090 | EU056953 |  |
| *C. eufulmineus* Rob. Henry | *Quercus humilis,* La Vall d’en Bas, Gerona, Spain | Arangu-Cort-03102001 | EF014267^c^ |  |
|  | *Fagus sylvatica* and *Quercus* sp., Ebringen (Schönberg), Germany | TUB 012780 | EF014256^c^ |  |
|  | *Fagus sylvatica, Carpinus betulus*, and *Quercus* sp., Heugrumbach, Germany | TUB 012782 | EF014258^c^ |  |
|  | *Fagus sylvatica*, *Carpinus* *betulus*, and *Quercus* sp., Heugrumbach, Germany | TUB 012777 | EF014260^c^ |  |
|  | *Fagus sylvatica, Carpinus* *betulus*, and *Quercus* sp., Heugrumbach, Germany | TUB 012781 | EF014259^c^ |  |
|  | *Fagus sylvatica* and *Quercus* sp., Saalburg, Germany | TUB 012778 | EF014261^c^ |  |
|  | *Fagus sylvatica*, Buir (Eifel), Germany | TUB 011426 | AY174847^a^ |  |
|  | *Fagus sylvatica*, Schopfheim, Germany | TUB 012779 | EF014257^c^ | EU088226 |
|  | *Quercus faginea,* Zigotia, Álava, Spain | Arangu-Cort-00/27 | EF014268^c^ |  |
| *C. flavaurora* M.M. Moser & McKnight | *Picea engelmannii* and *Pinus contorta*, Wyoming: Teton Co., Fourmile Meadow, USA | JFA 9913 | EU056946 |  |
| *C. flavobulbus* Ammirati & M.M. Moser | *Quercus* *vacciniifolia, Q. chrysolepis,* *Lithocarpus* *densiflora*, *Arbutus menziesii, Pseudotsuga* *menziesii*, and *Pinus* sp., California: Del Norte Co., Danger Point, USA | JFA 11826 | EU057017 |  |
| *C*. *flavovirens* Rob. Henry | *Fagus sylvatica*, Buir (Eifel), Germany | TUB 011454 | AY174841^a^ |  |
|  | *Fagus sylvatica*, Ebringen (Schönberg), Germany | TUB 012712 | EU057034 | EU088212 |
| *C. frondosophilus* Bidaud |  | PC0090401 Typus | EU655667 |  |
| *C*. *fulvocitrinus* Jul. Schäff. ex Brandrud | *Fagus sylvatica* and *Picea abies*, Kalborn, Germany | TUB 012713 | EU056958 | EU088201 |
|  | *Fagus sylvestris* and *Quercus* sp., Oos, Rheinland-Pfalz, Germany | TUB 011434 | AY174828^a^ |  |
| *C. glaucescens* (Jul. Schäff.) M.M. Moser | *Quercus ilex*, Villarcayo, Burgos, Spain | Arangu-Cort-02110102 | EU655656 |  |
|  | *Quercus* sp., *Carpinus betulus*, and *Tilia* sp., Freyburg/Unstrut, Germany | TUB 011655 | EU655681 | EU655653 |
| *C. guttatus* Rob. Henry | *Picea engelmannii* and *Pinus contorta*, Wyoming: Teton Co., Fourmile Meadow, USA | JFA 9942 | EU057063 |  |
|  | *Picea engelmannii,* Wyoming: Teton National Park, Taggart Lake, USA | JFA 12399 | EU056994 |  |
|  | *Picea engelmannii*, *Pinus contorta*, and *Abies lasiocarpa*, Wyoming: Fremont Co., Brooks Lake, USA | JFA 12412 | EU056993 |  |
| *C*. *haasii* (M.M. Moser) M.M. Moser | *Picea abies* and *Pinus sylvestris*, Nagold-Haiterbach, Germany | TUB 012714 | EU056986 |  |
|  | *Picea abies* and *Abies alba*, Flözlingen, Germany | TUB 012715 | EU056985 |  |
|  | *Picea abies*, Oberjoch, Germany | TUB 011858 | AY669561^b^ | EU088208 |
| *C*. *humolens* Brandrud | *Fagus sylvatica* and *Quercus* sp., Eschweiler, Germany | TUB 011427 | AY174852^a^ |  |
|  | *Fagus sylvatica*, Pfaffenweiler, Germany | TUB 012722 | EU056954 | EU088222 |
|  | *Fagus sylvatica*, Ebringen (Schönberg), Germany | TUB 012723 | EU056955 |  |
| *C. insignibulbus* Bidaud & Moënne-Locc. | *Corylus avellana*, Freyburg/ Unstrut, Germany | TUB 011656 | EU655660 |  |
|  | *Fagus sylvatica*, Gersheim, Germany | TUB 011657 | EU655673 | EU655639 |
| *C. ionodactylus* Knutsson & Soop | *Corylus* *avellana*, Himmelsberga, Långlöt, Öland, Sweden | S: F41803 | EU057050 |  |
| *C. laberiae* (in prep.) | *Abies alba* (and *Picea abies*?), Weißholz, Göschweiler, Germany | TUB 011889 | AY669563^b^ |  |
|  | *Abies alba*, Weißholz, Göschweiler, Germany | TUB 011658 | EU655663 | EU655636 |
| *C. langeorum* Frøslev & T.S. Jeppensen | *Fagus sylvatica* and *Abies alba* Ebringen (Schönberg), Germany | TUB 012711 | EU056996 | EU655643 |
| *C. largentii* Ammirati & M.M. Moser | *Quercus garryana*, *Q. chrysolepis*, *Lithocarpus* *densiflora*, *Arbutus menziesii,* and *Pseudotsuga* *menziesii*, California: Humboldt Co., Boise Creek Campground, USA | JFA 11875  Isotype | EU057059 |  |
| *C*. *lilacinovelatus* Reumaux & Ramm | *Fagus sylvatica*, Ebringen (Schönberg), Germany | TUB 012736 | EU056959 |  |
|  | *Fagus sylvatica*, Sulz-Ernet, Germany | TUB 012737 | EU056960 |  |
|  | *Fagus sylvatica*, Schönberg, Germany | TUB 012719 | EU056961 | EU088205 |
|  | *Quercus ilex* and *Quercus faginea*, Barrasa de Mena, Burgos, Spain | Arangu-Cort-03102503 | EU057055 |  |
|  | *Fagus sylvatica* and *Quercus* sp., Lahr-Sulz, Germany | TUB 011659 | EU655657 |  |
|  | *Carpinus betulus*, Freyburg/ Unstrut, Germany | TUB 011660 | EU655668 |  |
| *C. majusculus* Kühner (= *C. alcalinophilus* Rob. Henry ss. Brandrud et al.) | *Fagus* *sylvatica* and *Carpinus betulus*, Taching-St.-Coloman, Germany | TUB 011433 | AY174837^a^ |  |
|  | *Corylus* *avellana*, Öland, Sweden | KS-CO 172 | EF014263^c^ |  |
|  | *Quercus pyrenaica,* Zigotia, Álava, Spain | Arangu-Cort-03103002 | EF014271^c^ |  |
|  | *Carpinus betulus*, Freyburg/Unstrut, Germany | TUB 011661 | EU655682 | EU655654 |
| *C*. *meinhardii* Bon | *Picea abies*, Oberjoch, Germany | TUB 011390 | AY174839^a^ |  |
|  | *Picea abies*, Oberjoch, Germany | TUB 011443 | AY174840^a^ |  |
| *C. molochinus* Bidaud & Ramm | *Quercus* sp. and *Carpinus betulus,* Freyburg/Unstrut, Schweigenberg, Germany | TUB 011662 | EU655662 |  |
|  | *Quercus ballota*, *Pinus sylvestris* and *P. nigra*, Mas d’Arrufat, La Carcellera, Morella, Spain | TUB 011663 | EU655664 | EU655649 |
| *C. montensis* Bidaud |  | PC0090399 Typus | EU655666 |  |
| *C. murellensis* Gut., Ballara, J.A. Cadiñanos, Palazón & Mahiques | *Quercus ballota*, *Pinus sylvestris* and *P. nigra*, Mas d’Arrufat, La Carcellera, Morella, Spain | Arangu-Cort 05102702b  Syntypus | EU655659 | EU655647 |
|  | *Quercus ballota*, *Pinus sylvestris* and *P. nigra*, Mas d’Arrufat, La Carcellera, Morella, Spain | TUB 011680 | EU684533 |  |
| *C. napus* Fr. | *Picea abies,* St. Blasien-Dachsberg, Germany | TUB 012717 | EU057067 |  |
|  | *Picea abies* forest, Mockfjärd, Dalarna, Sweden | S: F44393 | EU057069 | EU088232 |
| *C. natalis* D. Antonini & M. Antonini | *Quercus* sp., Freyburg/Unstrut, Germany | TUB 011664 | EU655661 |  |
|  | *Quercus ilex* ssp. *ballota*, Jaén, Gibralberca, Spain | TUB 011678 | EU655678 | EU655648 |
| *C*. *nymphicolor* Reumaux | *Fagus sylvatica*, Pfaffenweiler, Germany | TUB 011866 | AY669566^b^ | EU655642 |
|  | *Fagus sylvatica*, Ebringen (Schönberg), Germany | TUB 012718 | EU056963 |  |
| *C. ochraceopallescens* Moënne-Locc. & Reumaux | *Fagus sylvatica*, Karlstadt, Germany | TUB 012773 | EU057077 |  |
|  | *Fagus sylvatica*, *Carpinus* *betulus*, and *Quercus* sp., Heugrumbach, Germany | TUB 012774 | EU057074 | EU655635 |
| *C*. *odoratus* (Joguet ex M.M. Moser) M.M. Moser | *Fagus sylvatica* and *Quercus* sp., Eschweiler, Germany | TUB 011438 | AY174836^a^ |  |
| *C*. *odorifer* Britzelm. | *Picea abies*, Oberjoch, Germany | TUB 011383 | AY174817^a^ |  |
|  | *Picea abies*, Oberjoch, Germany | TUB 012720 | EU057052 | EU088215 |
| *C*. *olearioides* Rob. Henry | *Quercus* sp., Mürlenbach (Eifel), Germany | TUB 012721 | EF014264^c^ | EU088224 |
| *C. olivascentium* Rob. Henry (= *C. xanthochlorus* Rob. Henry) | *Corylus avellana, Quercus* sp., and *Tilia* sp.*,* Halltorps Hage, Schweden | S: F44373 | EU057047 |  |
|  | *Fagus sylvatica* and *Quercus robur,* Urkabuztaiz, Álava, Spain | Arangu-Cort-03102901 | EU057068 |  |
|  | *Fagus sylvatica*, *Carpinus* *betulus*, and *Quercus* sp., Heugrumbach, Germany | TUB 012775 | EU057078 | EU655645 |
| *C*. *osmophorus* P.D. Orton | *Fagus sylvatica*, Loogh (Eifel), Germany | TUB 011445 | AY174816^a^ |  |
|  | *Fagus sylvatica*, Loogh (Eifel), Germany | TUB 011399 | AY174815^a^ |  |
|  | *Fagus sylvatica*, Tuttlingen-Esslingen, Germany | TUB 012724 | EU056990 | EU088210 |
| *C. parafulmineus* Rob. Henry | *Pinus sylvestris,* Roncal, Navarra, Spain | Arangu-Cort-0301201 | EF014269^c^ |  |
| *C. piceae* Frøslev, T.S. Jeppensen & Brandrud | *Picea abies*, Oberjoch, Germany | TUB 011385 | AY174842^a^ |  |
|  | *Picea abies*, Hinterstein, Germany | TUB 012691 | EU056956 | EU088207 |
| *C. platypus* (M.M. Moser) M.M. Moser | *Fagus sylvatica*, Pfaffenweiler, Germany | TUB 012733 | EU056972 |  |
|  | *Fagus sylvatica*, Sulz-Ernet, Germany | TUB 012734 | EU056971 |  |
|  | *Corylus avellana*, Wehr-Hasel, Germany | TUB 012735 | EU056973 |  |
|  | *Fagus sylvatica*, Sulz, Germany | TUB 011665 | EU660939 |  |
| *C. praetermissus* Bergeron & Reumaux | *Quercus ilex* ssp. *rotundifolia* and *Pinus halepensis*, Castelló, Morella, Mas d’Arrufat, Spain | MES-4312 | EU684535 |  |
|  | *Quercus ilex* ssp. *rotundifolia*, Castelló, Morella, Monte Palomita, Spain | MES-4294 | EU684534 |  |
| *C*. *prasinus* (Schaeff.: Fr.) Fr. | *Fagus* *sylvatica* and *Picea* *abies*, Nohn (Eifel), Germany | TUB 011431 | AY174835^a^ | EU088216 |
|  | *Fagus* *sylvatica* and *Pinus* *sylvestris*, Nohn (Eifel), Germany | TUB 012726 | EU057029 | EU088218 |
| *C*. *pseudoglaucopus* (Jul. Schäff. ex M.M. Moser) Nezdojm. | *Picea abies*, Tête de Ran, Switzerland | TUB 012730 | EU056950 | EU088223 |
|  | *Picea abies*, La Chaux-de-Fonds, Switzerland | TUB 012731 | EU056952 |  |
| (as *C*. *elotus* Fr.) | *Picea abies*, Trentino, Italy | IB 1999/0192 | EU056951 |  |
| *C. rapaceotomentosus* Delaporte & Eyssart. (=? *C. lutulentus* Jul. Schäff.) | *Fagus sylvatica*, Sielbeck, Germany | TUB 011666 | EU655655 |  |
|  | *Fagus sylvatica*, Ormesheim, Germany | TUB 011667 | EU655676 | EU655640 |
|  | *Carpinus betulus* and *Quercus* sp., Freyburg/ Unstrut, Germany | TUB 011668 | EU655679 |  |
| *C. roseobulbus* M.M. Moser | *Quercus garryana*, California: Del Norte Co., Gasquet Flat, USA | JFA 11850  Isotype | EU056982 |  |
|  | *Quercus garryana*, California: Del Norte Co., Gasquet Flat, USA | JFA 11838 | EU057062 |  |
| *C*. *rufoolivaceus* (Pers.: Fr.) Fr | *Fagus* *sylvatica* and *Quercus* sp*.*, Nohn (Eifel), Germany | TUB 011405 | AY174845^a^ | EU088219 |
|  | *Fagus* *sylvatica*, Buir (Eifel), Germany | TUB 012739 | EU057030 |  |
| *C*. *saporatus* Britzelm. ss. Brandrud et al. | *Fagus* *sylvatica* and *Quercus* sp., Eschweiler, Germany | TUB 011410 | AY174846^a^ | EU088220 |
|  | *Fagus sylvatica*, Bleichröder Berge, Germany | TUB 011880 | AY669570^b^ |  |
| *C. saxamontanus* Fogel | *Abies lasiocarpa, Picea engelmannii*, and *Pinus contorta*, Wyoming: Fremont Co., Brooks Lake, USA | MTS-4444 | EU057027 |  |
|  | *Abies amabilis, Larix occidentalis, Picea engelmannii, Pinus ponderosa*, *P. monticola,* and *Pseudotsuga menziesii,* Washington: Kittitas Co., Liberty-Swauk Pass area, USA | ST-97-166-11 | EU057026 |  |
|  | *Abies* sp.*, Larix occidentalis*, *Picea engelmannii,* and *Pinus contorta,* Washington: Kittitas Co., Peoh Point, USA | BW 5-31-04-1 | EU057028 | EU088216 |
| *C. selandicus* Frøslev & T.S. Jeppensen | *Quercus ilex,* Villarcayo, Burgos, Spain | Arangu-Cort-02/31 | EU057070 |  |
| *C*. *sodagnitus* Rob. Henry | *Fagus sylvatica and Quercus* sp*.*, Eschweiler, Germany | TUB 011428 | AY174829^a^ |  |
|  | *Fagus sylvatica* and *Quercus* sp*.*, Weyer, Germany | TUB 012740 | EU056970 |  |
|  | *Quercus ilex, Sardinia, Italy* | TUB 012725 | EU057076 | EU088200 |
| *C*. *splendens* Rob. Henry | *Fagus sylvatica*, Gerolstein-Gees, Germany | TUB 011411 | AY174833^a^ |  |
|  | *Fagus sylvatica*, Tuttlingen-Esslingen, Germany | TUB 011432 | AY174832^a^ |  |
|  | *Fagus sylvatica*, Tübingen, Schönbuch, Germany | TUB 012741 | EU057012 | EU088227 |
| *C. splendidior* Bidaud |  | PC0090402 Typus | EU655665 |  |
| *C. splendificus* Chevassut & Rob. Henry | *Quercus* sp., Badenweiler, Germany | TUB 011669 | EU655670 | EU655650 |
|  | *Quercus ilex* subsp. *ballota*, Jaén, Gibralberca, Spain | TUB 011679 | EU655677 |  |
| *C*. *suaveolens* Bataille & Joachim | *Fagus sylvatica*, Pfaffenweiler, Germany | TUB 012742 | EU056991 |  |
|  | *Tilia* sp*.*, St. Margarethen, Austria | TUB 011876 | AY669574^b^ | EU088234 |
| *C. subgracilis* Möenne-Locc. | *Quercus ilex,* Villarco, Burgos, Spain | Arangu-Cort-02/30 | EU057054 |  |
|  | *Picea abies*, *Abies alba* and *Pinus sylvestris*, Waldhausen, Kirnbergsee, Germany | TUB 011670 | EU655658 | EU655646 |
| *C. sublilacinopes* Bidaud, Moënne-Locc. & Reumaux | *Fagus sylvatica*, Tuttlingen-Esslingen, Germany | TUB 012697 | EU056988 |  |
|  | *Fagus sylvatica*, Gersheim, Germany | TUB 011671 | EU655674 | EU655652 |
| *C. subpurpureophyllus* A.H. Sm. var. *sulphureovelatus* M.M. Moser | *Tsuga heterophylla* and *Pseudotsuga menziesii*, Oregon: Lincoln Co., Fogarty Creek State Park, USA | JFA 11723 | EU057016 |  |
| *C*. *sulfurinus* Quél. | *Picea abies*, *Pinus* sp. and *Abies alba*, Hornberg, Germany | TUB 011908 | AY669572^b^ |  |
|  | *Picea abies* and *Pinus sylvestris*, Jerischach, Austria | TUB 012743 | EU056995 | EU088213 |
| *C. verrucisporus* Thiers & A.H. Sm. | *Pinus ponderosa, Pseudotsuga* *menziesii*, and *Larix occidentalis,* Oregon: Jefferson Co., Metolius River Basin, USA | OSC-51019 | EU057043 |  |
|  | *Pinus lambertiana* and *Abies concolor*, California: Sierra Co., Steele Gulch, USA | OSC-69621 | EU057045 |  |
|  | *Pinus contorta, P. lambertiana, P. ponderosa* and *Abies concolor*, Oregon: Klamath Co. North of Scott Creek, USA | OSC-74248 | EU057044 | EU088209 |
|  | *Pinus contorta, P. lambertiana, P. ponderosa* and *Abies concolor*, Oregon: Klamath Co., north of Pearce Point, USA | OSC-74303 | EU057042 |  |
| *C. vesterholtii* Frøslev & T.S. Jeppesen | *Carpinus betulus,* Freyburg/Unstrut, Germany | TUB 011672 | EU660941 | EU655637 |
|  | *Quercus* sp., and *Carpinus betulus*, Freyburg/ Unstrut, Germany | TUB 011673 | EU655686 |  |
|  | *Fagus sylvatica* and *Carpinus betulus*, Tengling-St., Bayern, Germany | TUB 011674 | EU655687 |  |
| *C. violaceipes* Bidaud & Consiglio | *Quercus suber* and *Quercus* sp., Caldana, Italy | TUB 012727 | EU057041 | EU088203 |
|  | *Fagus sylvatica*, Ebringen (Schönberg), Germany | TUB 012728 | EU057040 |  |
|  | *Fagus sylvatica* and *Quercus* sp., Eschweiler, Germany | TUB 011439 | AY174818^a^ |  |
|  | *Fagus sylvatica*, Ebringen (Schönberg), Germany | TUB 012729 | EU057039 |  |
|  | *Quercus suber* and *Quercus* sp., Caldana, Italy | TUB 011675 | EU655683 |  |
|  | *Quercus* sp., *Carpinus betulus*, and *Tilia* sp., Freyburg/ Unstrut, Germany | TUB 011676 | EU655688 |  |
| *C. viridirubescens* M.M. Moser & Ammirati | *Lithocarpus densiflora,* California: Mendocino Co., Road 408, USA | JFA 11817 | EU057007 |  |
|  | *Quercus garryana*, *Q. chrysolepis*, *Lithocarpus* *densiflora*, *Arbutus menziesii,* and *Pseudotsuga* *menziesii*, California: Humboldt Co., Boise Creek Campground, USA | JFA 11876 | EU057009 |  |
|  | *Lithocarpus densiflora*, and *Pseudotsuga menziesii*, California: Mendocino Co., County Road 408, USA | JFA 11895 | EU057008 |  |
| *C*. *xanthophyllus* (Cooke) Maire | *Fagus sylvatica*, Weyer, Germany | TUB 011453 | AY174826^a^ |  |
|  | *Fagus sylvatica*, Weyer, Germany | TUB 011457 | AY174827^a^ |  |
|  | *Fagus sylvatica* and *Quercus* sp., Schlechtberg, Germany | TUB 012744 | EU057023 | EU088217 |
| *C*. *xanthophyllus* (Cooke) Maire [*claroflavus* morph] | *Fagus sylvatica*, Dettenhausen, Germany | TUB 012745 | EU057071 |  |
| *Cortinarius* sp. EUR 1 | *Fagus sylvatica*, *Carpinus* *betulus*, and *Quercus* sp., Heugrumbach, Germany | TUB 012738 | EU057075 |  |
| *Cortinarius* sp. EUR 2 | *Fagus sylvatica*, Ormesheim, Germany | TUB 011677 | EU655675 | EU655651 |
| *Cortinarius* sp. USA 1 | *Arbutus menziesii, Lithocarpus* *densiflora*, *Quercus* *vacciniifolia, Q. chrysolepis,* *Pinus* sp., and *Pseudotsuga* *menziesii*, California: Del Norte Co., Danger Point, USA | JFA 11854 | EU056984 |  |
| *Cortinarius* sp. USA 2 | *Thuja occidentalis,* *Tsuga heterophylla*, *Pseudotsuga menziesii, Pinus ponderosa*, *Picea engelmannii,* and *Abies concolor,* Oregon: Wasco Co., Clear Creek Campground, USA | JFA 11646 | EU056962 |  |
| *Cortinarius* sp. USA 3 | *Quercus garrayana*, California: Del Norte Co., Gasquet Flat, USA | JFA 11847 | EU056964 |  |
| *Cortinarius* sp. USA 4 | *Picea engelmannii*, Wyoming: Teton National Park, Reid Mountain, USA | JFA 12417 | EU056980 |  |
|  | *Picea engelmannii* and *Pinus contorta*, Wyoming: Teton Co., Fourmile Meadow, USA | JFA 9914 | EU056979 |  |
| *Cortinarius* sp. USA 5 | *Arbutus menziesii, Lithocarpus* *densiflora*, *Quercus* *vacciniifolia, Q. chrysolepis,* *Pinus* sp., and *Pseudotsuga* *menziesii*, California: Del Norte Co., Danger Point, USA | JFA 11855 | EU056981 |  |
| *Cortinarius* sp. USA 6 | *Abies concolor,* *Picea engelmannii, Pinus ponderosa*, *Pseudotsuga menziesii,* and *Tsuga heterophylla*, Oregon: Wasco Co., Clear Creek Campground, USA | JFA 11649 | EU056977 |  |
| *Cortinarius* sp. USA 7 | *Quercus garryana*, California: Del Norte Co., Gasquet Flat, USA | JFA 11832 | EU057020 |  |
|  | *Quercus garryana*, California: Del Norte Co., Gasquet Flat, USA | JFA 11834 | EU057022 |  |
|  | *Quercus garryana*, California: Del Norte Co., Gasquet Flat, USA | JFA 11846 | EU057021 |  |
| *Cortinarius* sp. USA 8 | *Quercus garryana*, California: Del Norte Co., Gasquet Flat, USA | JFA 11849 | EU057031 |  |
| *Cortinarius* sp. USA 9 | *Quercus* *garryana*, California: Del Norte Co., Gasquet Flat, USA | JFA 11833 | EU057035 |  |
|  | *Quercus garryana*, California: Del Norte Co., Gasquet Flat, USA | JFA 11845 | EU057036 |  |
| *Cortinarius* sp. USA 10 | *Lithocarpus densiflora*, *Quercus chrysolepis*, and *Pseudotsuga menziesii,* California: Del Norte Co., Patrick Creek Campground, USA | JFA 11767 | EU057038 |  |
| *Cortinarius* sp. USA 11 | *Quercus douglasii* and *Q. wislizenii,* California: Yuba Co.: UC Sierra Foothill Research & Extension Center, USA | SRC-523 | EU057033 |  |
|  | *Quercus wislizenii,* California: Yuba Co.: UC Sierra Foothill Research & Extension Center, USA | SRC-608 | EU057032 |  |
| *Cortinarius* sp*.* USA 12 | *Tsuga heterophylla* and *Pseudotsuga menziesii*, Oregon: Clackamus Co., Bull Run, USA | JFA 11701 | EU057024 |  |
| *Cortinarius* sp. USA 13 | *Lithocarpus densiflora*, California: Del Norte Co., Big Flat Station, USA | JFA 11802 | EU057025 |  |
| *Cortinarius* sp. USA 14 | *Thuja occidentalis,* *Tsuga heterophylla*, *Pseudotsuga menziesii, Pinus ponderosa*, *Picea engelmannii,* and *Abies concolor,* Oregon: Wasco Co., Clear Creek Campground, USA *(as C. corrosus)* | JFA 11618 | EU056947 |  |
| *Cortinarius* sp. USA 15 | *Pseudotsuga menziesii*, *Abies grandis, A. procera*, and *Picea engelmannii*, Oregon: Wasco Co., Clear Creek Crossing Campground, USA | JFA 11619 | EU056949 |  |
| *Cortinarius* sp. USA 16 | *Abies nobilis/magnifica, Tsuga mertensiana*, and *Pinus contorta*, Oregon: Klamath Co., Crater Lake National Park, USA | OSC-81327 | EU056976 | EU088204 |
| *Cortinarius* sp. USA 17 | *Quercus garrayana*, California: Del Norte Co., Gasquet Flat, USA | JFA 11836 | EU057046 |  |
